# Personal Thermal Management by Radiative Cooling and Heating

**DOI:** 10.1007/s40820-024-01360-1

**Published:** 2024-03-13

**Authors:** Shidong Xue, Guanghan Huang, Qing Chen, Xungai Wang, Jintu Fan, Dahua Shou

**Affiliations:** 1 School of Fashion and Textiles, The Hong Kong Polytechnic University, Hung Hom, Kowloon, Hong Kong, 999077 People’s Republic of China; 2 Future Intelligent Wear Centre, The Hong Kong Polytechnic University, Hung Hom, Kowloon, Hong Kong, 999077 People’s Republic of China; 3 State Key Laboratory of Precision Electronic Manufacturing Technology and Equipment, Guangdong University of Technology, Guangzhou, 510006 People’s Republic of China; 4 Shanghai International Fashion Innovation Center, Donghua University, Shanghai, 200051 People’s Republic of China; 5 Research Centre of Textiles for Future Fashion, The Hong Kong Polytechnic University, Hung Hom, Kowloon, Hong Kong, 999077 People’s Republic of China; 6 Research Institute for Smart Energy, The Hong Kong Polytechnic University, Hung Hom, Kowloon, Hong Kong, 999077 People’s Republic of China

**Keywords:** Personal thermal management, Radiative cooling and heating, Thermal comfort, Dynamic thermoregulation

## Abstract

Maintaining thermal comfort within the human body is crucial for optimal health and overall well-being. By merely broadening the set-point of indoor temperatures, we could significantly slash energy usage in building heating, ventilation, and air-conditioning systems. In recent years, there has been a surge in advancements in personal thermal management (PTM), aiming to regulate heat and moisture transfer within our immediate surroundings, clothing, and skin. The advent of PTM is driven by the rapid development in nano/micro-materials and energy science and engineering. An emerging research area in PTM is personal radiative thermal management (PRTM), which demonstrates immense potential with its high radiative heat transfer efficiency and ease of regulation. However, it is less taken into account in traditional textiles, and there currently lies a gap in our knowledge and understanding of PRTM. In this review, we aim to present a thorough analysis of advanced textile materials and technologies for PRTM. Specifically, we will introduce and discuss the underlying radiation heat transfer mechanisms, fabrication methods of textiles, and various indoor/outdoor applications in light of their different regulation functionalities, including radiative cooling, radiative heating, and dual-mode thermoregulation. Furthermore, we will shine a light on the current hurdles, propose potential strategies, and delve into future technology trends for PRTM with an emphasis on functionalities and applications.

## Highlights

- This review delves into the intricate relationship between thermal models, function-oriented design principles, and practical applications in personal radiative thermal management (PRTM).
- It provides an in-depth discussion on design strategies for radiative cooling, heating, and dual-mode modulating textiles, offering practical insights for application.
- It offers a thorough examination of the prospects and challenges of PRTM textiles, proposing potential solutions and future directions for the field.

## Graphical abstract

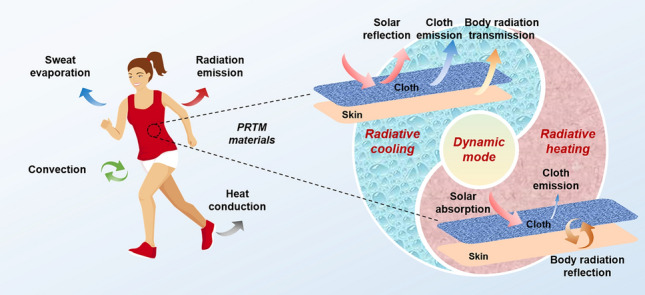

## Introduction

Thermal comfort, which is defined as “a state in which there are no driving impulses to correct the environment by the behaviors,” is crucial to human comfort, health, performance, and overall well-being. It is also interpreted as “the condition of mind that expresses satisfaction with the thermal environment” [1]. The human body’s thermal comfort zone was proposed in the standard ASHRAE 55-1992, which outlines suitable temperature and humidity ranges for summer and winter [2, 3]. Regulating thermal comfort is crucial for maintaining normal metabolism activities within the body’s stable temperature range of 36.0–37.3 °C [4]. Any deviations from this range, such as hyperthermia (core body temperature exceeding 37.5–38.3 °C) or hypothermia (core body temperature below 35.0 °C), can have adverse effects on physical and psychological well-being, and even pose a threat to life [5].

The increasingly high temperatures caused by global warming have presented urgent challenges to daily life. In 2023, exceptional heatwaves have been occurring globally, setting new temperature records. The discomfort stimulated by these extremely hot conditions can prompt a decline in productivity and efficiency, potentially provoking health complications for workers. The climate change particularly affects those who endure harsh or severe atmospheric environments for prolonged periods such as miners, firefighters, soldiers, athletes, construction workers, foundry workers, and agricultural laborers [6]. In the long run, the heatwaves and associated thermal discomfort can negatively affect the economy and quality of life [5, 7].

Furthermore, regulating thermal comfort is crucial for energy saving in building heating, ventilation, and air-conditioning (HVAC) systems, which account for approximately one-third of energy consumption in the commercial and residential sectors, and about 15.2% of total domestic primary energy usage according to the US Department of Energy [8]. Studies have shown that expanding the heating or cooling temperature set-point by 2 °C can result in approximately 20% energy savings for HVAC systems [9]. It is well-known that global warming and the energy crisis resulting from fossil fuel combustion and greenhouse gas emissions are two pressing challenges in the twenty-first century. Therefore, it is essential and promising to develop new strategies and technologies to regulate human thermal comfort, taking into account social development, personal health, and energy conservation.

**Fig. 1:**
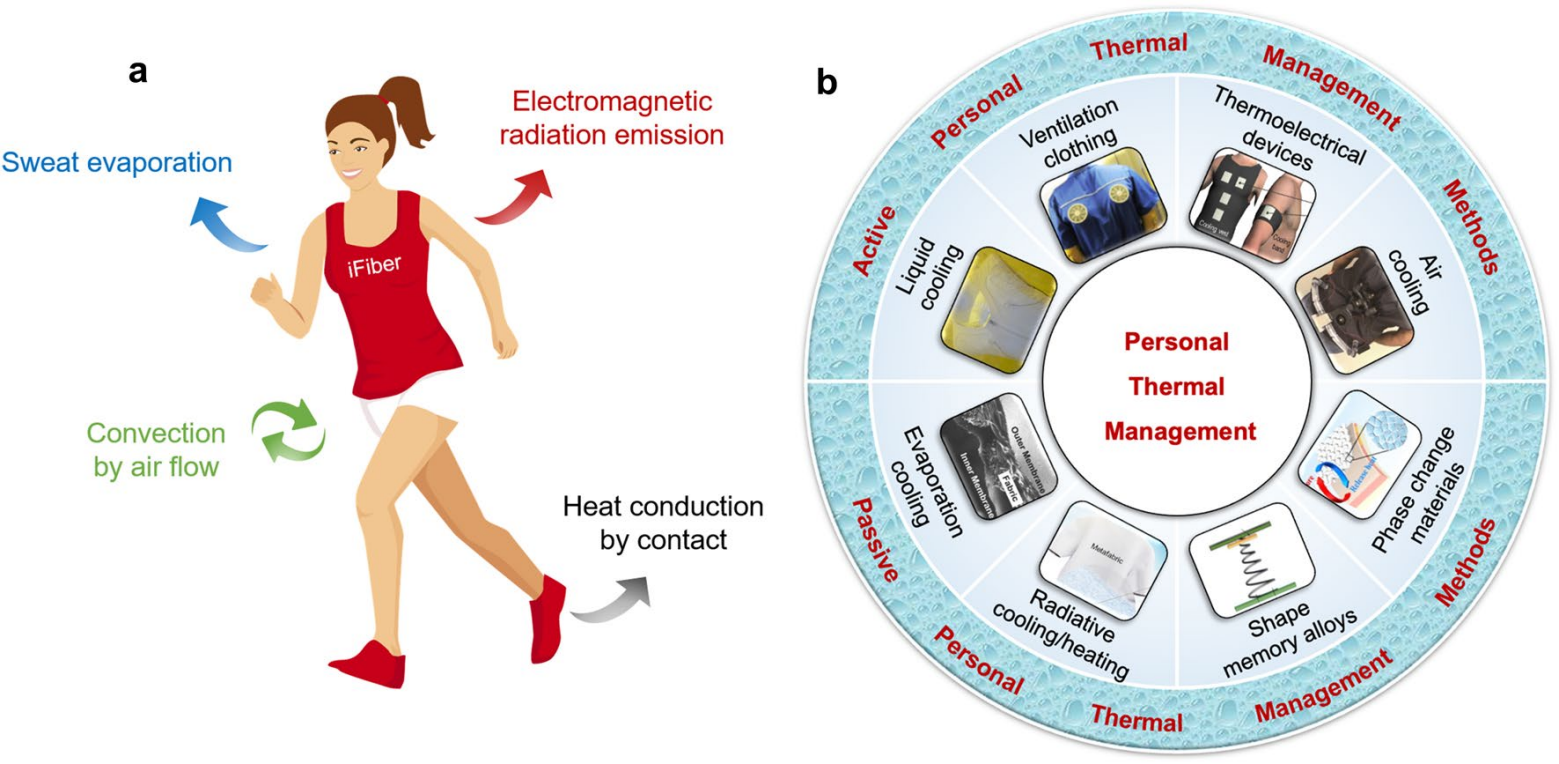
Heat dissipation of human body and main PTM methods. **a** Heat transfer mechanism of human body. **b** Categories of PTM strategies for thermal comfort [13–20]. Reproduced with permission. Copyright 2013, Elsevier. Copyright 2021, American Association for the Advancement of Science. Copyright 2019, Elsevier. Copyright 2013, Elsevier. Copyright 2008, Springer Nature. Copyright 2015, Elsevier. Copyright 2020, American Chemical Society

In order to maintain thermal comfort, the human body needs to balance the heat it produces with the heat it dissipates. Heat generated through physical activity is mainly dissipated through radiation, conduction, convection, and evaporation, as shown in Fig. 1a. The first three mechanisms of heat dissipation are based on dry heat transfer, which is influenced by the thermal and optical properties of the clothing. On the other hand, sweat evaporation from the fabric, which is a wet heat transfer process, depends on the permeability of the clothing material and the surrounding air velocity [10, 11]. The contribution of each heat dissipation route can vary depending on the circumstances. For example, radiation in the mid-infrared wavelength range is responsible for up to 50% of heat loss in a typical indoor environment. However, during intensive exercise or under higher air velocity conditions, most of the generated heat is dissipated through evaporation [12]. Normally, the human body can regulate thermal comfort through various thermoregulatory activities such as blood circulation, muscle movement, shivering, perspiration, and metabolic rate. However, the heat load on the human body can exceed its natural thermoregulatory capacity, leading to discomfort, heatstroke, or hypothermia in harsh environments or extreme climates like intense sunlight, hot environments, or storms. Since it is not possible to easily modulate the ambient environment due to operational restrictions, it is necessary to incorporate advanced textile materials into clothing or develop smart, wearable, and portable thermal regulation devices. These innovations can protect people from severe heat or cold strain and ensure their comfort and safety in challenging conditions.

Recent advancements in energy science and micro/nano-material fabrication technology have led to significant growth and progress in the field of PTM, which have greatly contributed to the regulation of thermal comfort and overall well-being [21]. PTM involves controlling and regulating localized heat and mass transfer between the skin, clothing, and the surrounding environment. This technique is highly energy-efficient and cost-saving compared to the HAVC systems designed for indoor spaces [22]. PTM aims to provide appropriate and personalized thermal dosage for individual occupants to ensure thermal comfort. On the other hand, HAVC systems are limited to indoor environments and are impractical and expensive for outdoor applications [23].

PTM combines advanced functional materials and emerging technology strategies and can be classified into different types. For example, PTM techniques can be categorized as off-body systems and on-body wearable devices, depending on how they interact with the human body during movement [24]. Additionally, PTM includes active, passive, and hybrid thermal regulation techniques based on whether an external energy source or auxiliary driving force is needed to circulate the coolant for heat removal [21]. Hybrid personal thermal systems combine at least two active or passive methods to regulate thermal comfort for the human body. As shown in Fig. 1b, active thermal regulation techniques include air cooling systems, liquid cooling systems, micro-fan ventilation systems, thermoelectrical systems, and Joule heat clothing. Passive thermal regulation techniques widely utilized in PTM include phase change material-based (PCM) systems, shape memory alloys-based (SMA) materials, conductive cooling systems, evaporation cooling systems, and radiative cooling/heating systems.

The working principles for thermal regulation and the individual characteristics for personal application are summarized in Table 1. Compared to other types of PTM, liquid cooling systems are known for their efficiency and larger heat capacity. However, they tend to be heavy, bulky, and not easily portable for outdoor environments [25]. In contrast, PTM systems with micro-fan ventilation devices, which are strategically placed in clothing regions where the human body generates heat rapidly and frequently, offer a lighter and more portable solution [26]. The thermoelectric system is typically based on the Peltier effect, which occurs when an electrical current passes through a circuit consisting of two different conductors joined together. The thermoelectric effect facilitates the conversion of electrical power into thermal energy [27, 28]. The thermoelectric module, with its unique characteristics, offers significant advantages, including excellent flexibility, high reliability, and wearability. Furthermore, it operates without vibrating parts, enabling direct energy conversion and eliminating the need for coolants [29]. However, it is important to note that thermoelectric modules typically exhibit a poor coefficient of performance across a wide temperature range [30]. Phase change materials (PCMs) are widely used as sources of energy storage in PTM [31]. These materials store energy through a phase change, and the stored energy can be retrieved and utilized for the required thermal regulation [32, 33]. The use of PCM-based thermal regulation systems has been verified to be safe for workers who are subject to high heat stress. However, the main limitation of using PCM is the low thermal conductivity of the materials used to fill the system. This low thermal conductivity can impede the rapid transfer of heat [34, 35]. Shape memory alloys (SMAs), such as Ni–Ti, are metallic compounds composed of two or more metals, which are often integrated into the inner layer of protective clothing. These alloys undergo a shape expansion when triggered by a specific actuation temperature, which creates air gaps between the clothing layers, hindering the transfer of heat to the human body [36, 37]. When the ambient temperature decreases, SMAs have the ability to regain their original shapes. One advantage of SMAs is that they do not require any battery or power source for their operation. However, incorporating SMAs into clothing can be challenging, as they are difficult to be bonded directly to the clothing material [38]. Heat conduction plays a significant role in heat transfer at the interface between the inner layer of fabric and the human body’s skin. Additionally, heat transfer inside the fabric primarily occurs through conduction. As a result, cooling garments require highly conductive materials to facilitate heat dissipation, while heating garments necessitate low conductive or thermally insulated materials to retain heat [39, 40]. Various coating materials, such as carbon nanotubes (CNTs) [41], graphene [42–44], and boron nitride [45, 46], are embodied in the clothing for improving thermal conductivity, but these coatings may be prone to cracking or damage after repeated wearing and washing [47]. In addition, it is important to pay attention to health-related issues of the coating materials for PTM. An evaporation cooling system primarily utilizes water or wet air as the coolant for removing heat, as water has a large latent heat capacity and is considered environmentally friendly. These unique advantages make it an ideal choice for cooling purposes in evaporation cooling systems [48, 49]. Membrane-based cooling garments and vacuum desiccant-based cooling garments are two popular types of evaporation coolers in PTM. Despite their advantages, such as efficient cooling, they have a tendency to degrade in performance and efficiency when exposed to prolonged sunlight. On the other hand, radiative cooling/heating has enormous potential for energy saving in achieving human body thermal comfort. This technique is passive in nature and offers excellent energy efficiency [50]. Human skin, with an emissivity of over 0.95, can be considered as a near-black radiating source. This characteristic makes it an exceptional emitter in the mid-infrared (MIR) range, with wavelengths typically falling between 7 and 14 μm [51]. Achieving heating or cooling in fabric materials through rational structure design, which involves regulating their optical properties such as emissivity, transmittance, and reflectance, has emerged as a popular research topic in recent years. This approach allows for automatic thermal regulation based on the ambient conditions, providing the desired level of comfort for the human body [52]. However, the white or silvery reflectance of these fabric surfaces may not be visually, and the restricted comfortable wearability of such materials is another drawback that hampers their widespread adoption [53]. Although various PTM methods and techniques have been developed and implemented in everyday life and industrial production, the selection of the appropriate method for regulating thermal comfort of the human body primarily depends on the specific requirements of the application scenario. Affecting factors such as heat capacity, portability, flexibility, weight, and safety play a crucial role in determining the optimal PTM solution.

**Table 1:** Comparison of different technologies for PTM

PTM technology	Active/passive	Advantages	Disadvantages
Air cooling garment	Active	Light weight and low energy consumption	Low capacity and challenges in being embodied into clothing
Liquid cooling garment	Active	Large heat capacity	Heavy, bulky fluid circulation systems
Ventilation clothing	Active	Enhanced evaporation	Bulky fluid circulation systems
Thermoelectrical devices	Active	Good flexibility, high reliability, no vibrating part, and direct energy conversion	Low efficiency in a wide temperature range and rising problems in phase segregation
Phase change materials	Passive	Large latent heat capacity	Inconvenient pretreatment, low thermal conductivity, and insufficient sweat removal
Shape memory alloys	Passive	Flexible shape transformation	Challenges in being embodied into clothing
Evaporation cooling	Passive	Huge latent heat capacity and environment friendliness	Over-cooling and less breathability
Radiative cooling/heating	Passive	High thermal regulation performance, cost-effective for energy saving, and flexible regulation	Weather dependence and limited wearable comfort

Among the PTM approaches mentioned above, radiative cooling/heating, also known as personal radiative thermal management, stands out as the most energy-efficient technique. This method achieves effective thermal regulation without the need for additional energy sources or consumption. Indeed, PRTM is expected to surpass conventional methods of cooling or heating. One of the significant advantages of PRTM over other types of PTM is the ability to flexibly control the heat transfer performance by optimizing the optical properties of clothing materials to meet individual requirements. This has the potential to provide “all-weather” thermal comfort satisfaction for users. The main design principle of PRTM-based textiles is to regulate the temperature near the human body’s skin, instead of the whole indoor space, which can expand the temperature set-point of HVAC systems and thus lower the energy consumption. Also, it can work in outdoor environments, even when people are directly exposed to sunlight. The purpose of PRTM is to enhance the thermal radiation dissipation of the human body under high-temperature weather and inhibit it in cold environments for thermal comfort regulation. Admittedly, it is more desirable and effective for controlling the human body’s temperature if the textile material can simultaneously modulate the optical behavior of sunlight and MIR waves emitted from the human body. However, the contribution factor of radiation to the total heat transfer, as well as the design of infrared (IR) optical properties of the clothing materials, are not considered for traditional textiles. In recent years, many efforts have been devoted to the design and fabrication of advanced textile materials from the benefit of the rapid development of energy materials and micro/nano-fabrication technologies. Many functional textiles have been used to successfully control the heat transfer performance between the human skin and the surrounding environment, and already commercially available in the market. This emerging and prosperous field has intrigued and attracted lots of researchers to focus on the design fundamentals of fiber nanostructure, fabrication technologies of advanced material, and engineering application of functional textiles, for the regulation of the human body’s thermal comfort. Additionally, PRTM technologies have been widely applied in other fields due to their excellent advantages, such as energy saving building, spacecraft cooling, energy harvesting, medical treatment and protection [54, 55].

In current literature, numerous research studies and reviews on PTM have been published. These primarily focus on the development of innovative functional textile materials, artificial skin, wearable sensors, and portable devices [56, 57]. The impact of these advanced materials and techniques on the thermal regulation efficiency of the human body, as well as their potential for energy savings in buildings, has been evaluated and projected. For example, Peng et al. [58] summarized the radiation- and conduction-controlled textiles, active cooling/heating textiles, as well as some smart responsive textiles with PCMs and dynamic structure changes for PTM. Similarly, Lei et al. [59] provided a comprehensive summary of the research advancements in the realm of advanced clothing, with a particular emphasis on those that primarily facilitate the dissipation of human body heat through radiation and conduction. They also engaged in a discussion on adaptive clothing, encompassing dual-mode and responsive textiles. Hu et al. [23] conducted a review on the emerging materials, strategies, and devices used in PTM, with a particular focus on their thermal functions. Ma et al. [24] provided a comprehensive overview of the human body’s thermoregulatory system, as well as the advantages and disadvantages associated with various technologies, including off-body near-range energy systems and on-body wearable textiles and devices. Sajjad et al. [21] summarized different types of active, passive, and hybrid cooled garments, along with a discussion on their respective applications, advantages, and limitations. Farooq et al. [60] also introduced advanced textiles of PTM for solving the above issues. It is evident that the majority of existing reviews primarily concentrate on offering a general and broad categorization and comparison of various advanced functional textiles used in PTM for managing thermal comfort in the human body. However, there is a scarcity of specific reviews that thoroughly discuss the subject of PTM via radiation or PRTM. Liang et al. [61] and Huang et al. [62] have specifically summarized typical radiative cooling materials and explored their diverse commercial applications in personal thermoregulation, as well as other fields such as energy-efficient buildings, solar cell cooling, and water harvesting. In 2021, Zhu et al. [63] discussed textile materials for PRTM in terms of the differences in radiation sources between indoor and outdoor thermal environments. In 2023, He et al. [64] summarized the principles of infrared radiative modulation and then demonstrated three corresponding design strategies for infrared radiative modulating textiles in terms of radiative cooling, radiative insulation, and Janus radiative textiles.

While there has been an increasing focus on PRTM, existing reviews have primarily concentrated on the overall development of textiles from a material fabrication perspective. Furthermore, comprehensive reviews that encapsulate the intricate relationship between thermal models, function-oriented design principles, and practical applications in PRTM are scarce. PRTM has seen a significant surge in development in recent years due to its high thermal performance and regulatory flexibility, showcasing a promising future with potential compared to other PTM techniques. This review, therefore, sets itself apart by specifically focusing on PTM related to radiative cooling and heating. We aim to present a systematic review that encompasses the mechanistic models of human body heat dissipation, the fundamental design principles of textile materials, the fabrication technology of structures, and the evaluation of regulatory performance. In Sect. 2, we will introduce the heat transfer model of the human body and discuss and summarize the regulation principles related to radiation heat transfer for PRTM based on radiation heat transfer theory. Sections 3–4 focus on advanced textile materials for PRTM. We will introduce and summarize these materials in three specific categories: radiative cooling materials, radiative heating/warming materials, and smart/dynamic textile materials capable of providing both cooling and heating functionalities.

## Radiation Heat Transfer Models and Regulation Principles

In this section, we will initially introduce and delve into the methods of heat dissipation between the human body and its environment, with a particular focus on radiative heat transfer models. Building upon these theories, we will systematically summarize the regulatory principles for different material types and structures in relation to their functionalities. Additionally, we will discuss the current methods used to evaluate personal radiative heat transfer performance.

### Heat Transfer Models of Human Body

**Fig. 2:**
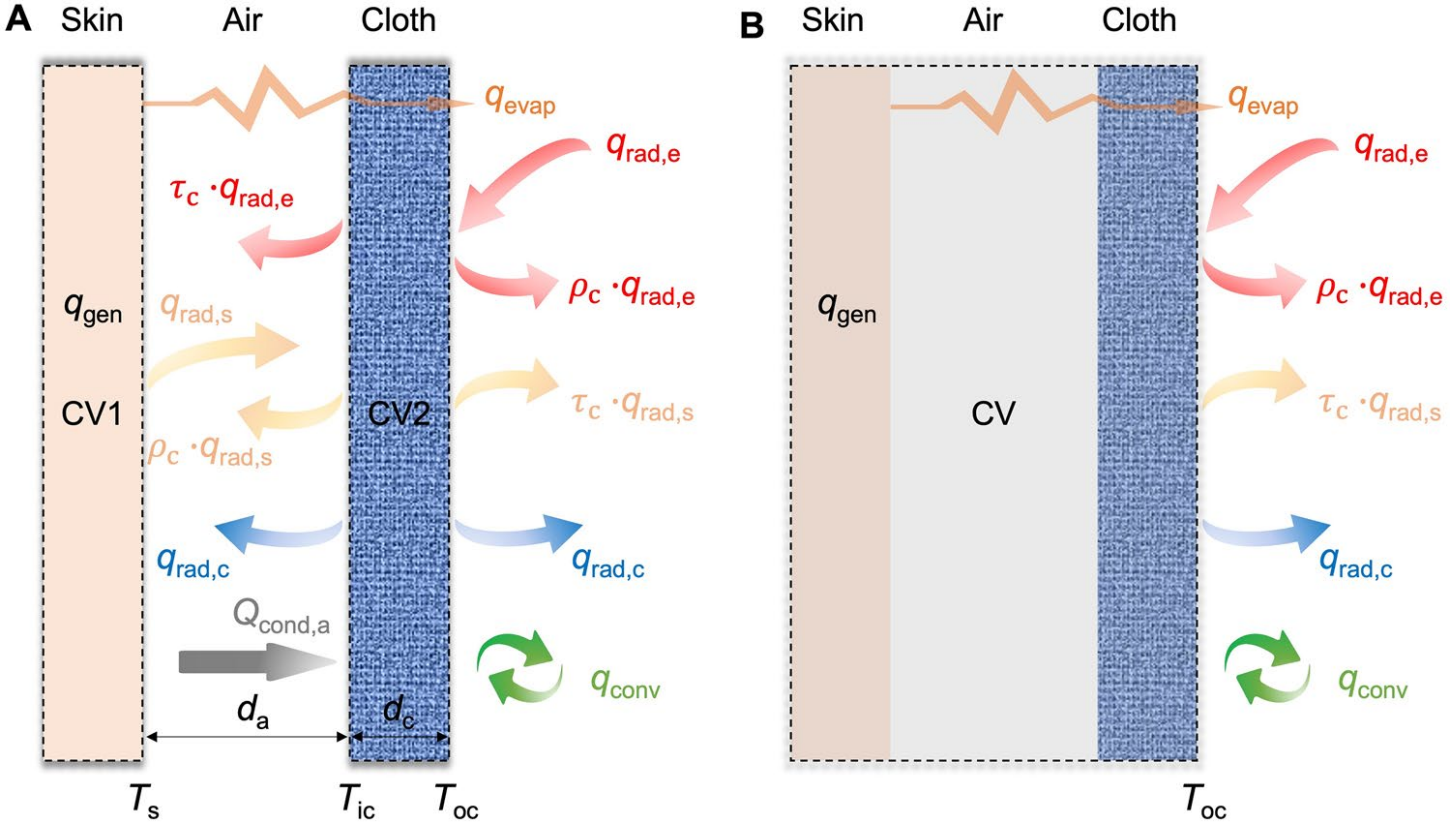
Schematic of the radiation heat transfer model for PRTM. **a** The skin and the cloth are considered as two separate control volumes. **b** The skin and the cloth are considered as a single unified control volume

The skin temperature is the competition result of the heat input generated from the metabolism or the ambient surroundings, and the heat dissipation through conduction, convection, sweat evaporation, and radiation. The heat transfer process from the human body to the ambient environment can be depicted in detail by adopting a 1D steady-state heat transfer model, considering the human body and the cloth as two individual control volumes, as demonstrated in Fig. 2a. The conduction between the skin and the cloth, and inside the cloth, the convection between the cloth and the ambient environment, and the radiation emitted from the human body, the cloth, and the ambient environment are included in this model. Specifically, the heat transfer equations for the two individual control volumes are expressed as follows by applying the energy balance theory.

For the human body:

1 $${q}_{\text{gen}}+{q}_{\text{rad,c}}+{\tau }_{{\text{c}}}\cdot {q}_{\text{rad,e}}-(1-{\rho }_{\text{c}})\cdot {q}_{\text{rad,s}}-{q}_{\text{cond,a}}-{q}_{\text{evap}}=0$$

For the cloth:

2 $$(1 - \rho_{{\text{c}}} - \tau_{{\text{c}}} ) \cdot q_{{\text{rad,e}}} + (1 - \rho_{{\text{c}}} - \tau_{{\text{c}}} ) \cdot q_{{\text{rad,s}}} + q_{{\text{cond,a}}} - 2 \cdot q_{{\text{rad,c}}} - q_{{{\text{conv}}}} = 0$$

where *q*_gen_ is the heat generation rate per unit area; *q*_rad,c_, *q*_rad,e_, and *q*_rad,s_ are the radiative heat flux from the cloth, the ambient environment, and the skin, respectively; *q*_evap_ is the evaporation heat flux from the skin to the environment; *q*_cond,a_ is the conductive heat flux between the skin and the cloth; *q*_conv_ is the convective heat flux from the cloth to the ambient environment [24]. ${\tau }_{{\text{c}}}$ and ${\rho }_{{\text{c}}}$ are the transmittance and reflectance of the cloth, respectively. According to Kirchhoff’s law, ${\varepsilon }_{{\text{c}}}$+${\tau }_{{\text{c}}}$+${\rho }_{{\text{c}}}$=1, where ${\varepsilon }_{{\text{c}}}$ is the emissivity of the cloth.

The conduction, convection, and radiation heat flux terms can be calculated as follows by Fourier’s law, Newton’s law of cooling law, and Stefan–Boltzmann law, respectively.

3 $$q_{{{\text{cond}},{\text{a}}}} = k_{{\text{a}}} \frac{{T_{{\text{s}}} - T_{{{\text{ic}}}} }}{{t_{{\text{a}}} }}$$

4 $$q_{{{\text{conv}}}} = h \cdot (T_{{{\text{oc}}}} - T_{{\text{e}}} )$$

5 $$q_{{\text{rad,s}}} = \sigma T_{{\text{s}}}^{{4}}$$

6 $$q_{{\text{rad,s}}} = \sigma T_{{\text{s}}}^{{4}}$$

7 $$q_{{\text{rad,c}}} = \varepsilon_{{\text{c}}} \sigma \cdot \left( {\frac{{T_{{{\text{ic}}}} + T_{{{\text{oc}}}} }}{2}} \right)^{4}$$

where *T*_s_, *T*_ic_,* T*_oc_, and *T*_e_ are temperatures of the skin, the inner cloth surface, the outer cloth surface, and the ambient, respectively; *k*_a_ is the thermal conductivity of air; *t*_a_ is the air gap thickness; *h* is the convective heat transfer coefficient; *σ* is the Stefan–Boltzmann constant equal to 5.67 × 10^−8^ W m^−2^ K^−4^.

**Fig. 3:**
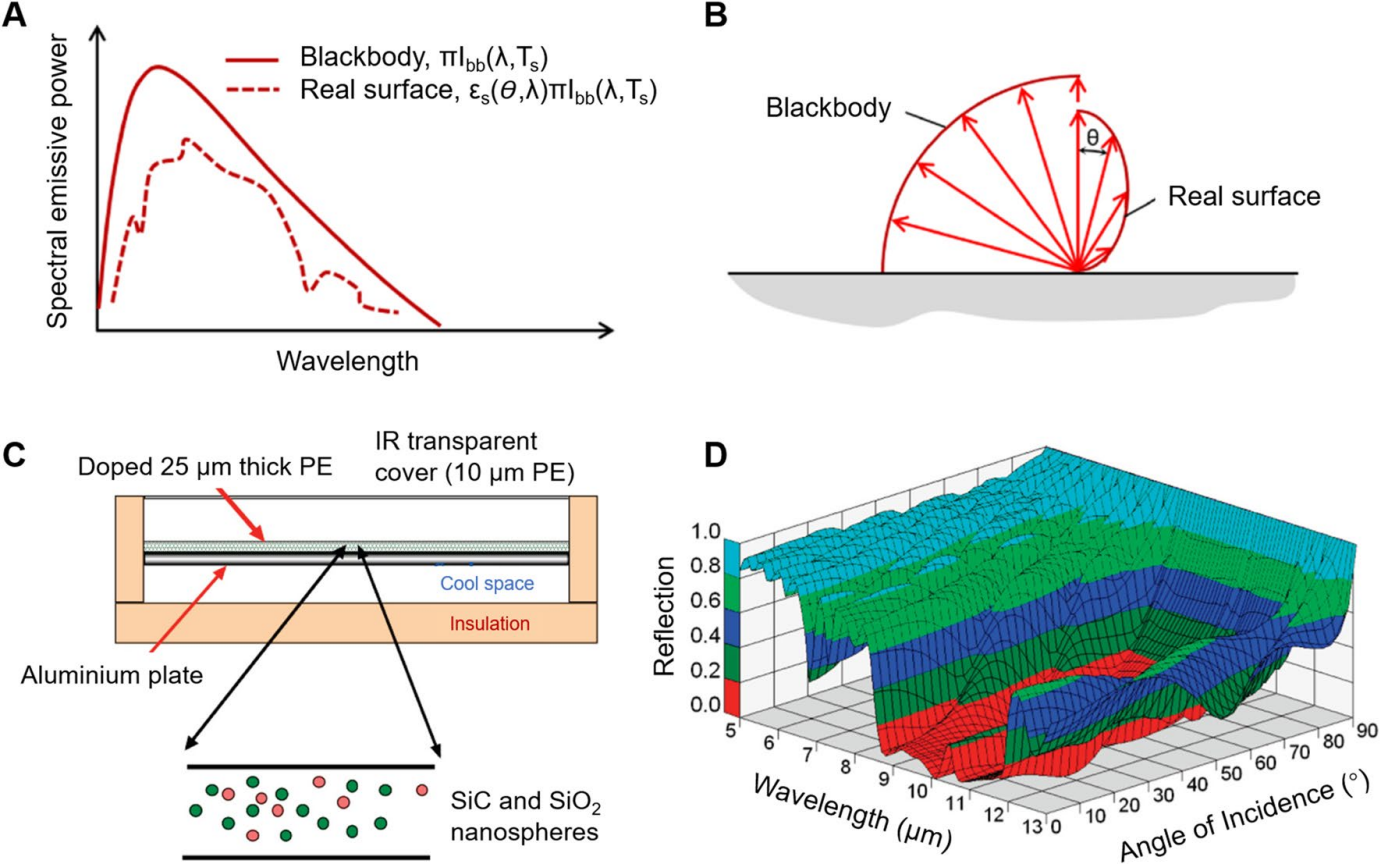
Fundamentals of radiative emissivity from a surface. **a** Spectral distribution of blackbody and real surface emission. **b** Directional distribution of blackbody and real surface emission [65]. Reproduced with permission. Copyright 2019, AIP Publishing. **c** A surface of doped polyethylene foil that contains SiC and SiO_2_ nanoparticles on the aluminum. **d** Reflectance as a function of wavelength and angle of incidence for doped polyethylene foil on aluminum [66]. Reproduced with permission. Copyright 2010, American Chemical Society

If the human body and the cloth are considered as a single control volume, the steady-state energy balance between the fabric and the ambient environment can be expressed as follows when the fabric is exposed to solar radiation, as shown in Fig. 2b.

8 $${k}_{{\text{c}}}\frac{{\text{d}}T}{{\text{d}}x}=\sigma {\varepsilon }_{\text{c}}\cdot \left({T}_{\text{oc}}^{4}-{T}_{\text{e}}^{4}\right)+h\cdot ({T}_{\text{oc}}-{T}_{\text{e}})-(1-{\rho }_{\text{c}})\cdot {q}_{\text{rad,e}}+{q}_{\text{evap}}$$

where *k*_c_ is the thermal conductivity of the cloth. The emissivity of the cloth surface (${\varepsilon }_{{\text{c}}}$) is usually assumed to be independent of the surface temperature and is mainly determined by the spectral wavelength (Fig. 3a) and angular direction (Fig. 3b), indicating that the emissivity for a specific material may have different values at a given wavelength or a given direction [65]. For example, the surface of doped polyethylene foil on the aluminum (Fig. 3c) showed a low reflectance (high emissivity) at a small angle of incidence, and the reflection would be closed to one as the angle of incidence approached to 90° [66] (Fig. 3d). For most materials, the surface emissivity usually remains relatively stable and high at an angle of incidence smaller than 60° [65, 67, 68]. Except for the spectral and wavelength dependence emissivity (${\varepsilon }_{{\text{c}}}$), the total hemispherical emissivity (${\overline{\varepsilon }}_{{\text{c}}}$) is usually used and defined as the total radiation energy emitted over all wavelengths and in all directions.

9 $$\overline{\varepsilon }_{{\text{c}}} = \frac{{\int {\cos \theta {\text{d}}\Omega \int_{0}^{\infty } {I_{{{\text{bb}}}} (\lambda ,T_{{{\text{oc}}}} )\varepsilon_{{\text{c}}} (\Omega ,\lambda )} } {\text{d}}\lambda }}{{A\sigma T_{{{\text{oc}}}}^{4} }}$$

where Ω is the solid angle between the direction of radiation and normal to the surface (Fig. 3b), *λ* is the wavelength of radiation emitted from the surface, ${\varepsilon }_{{\text{c}}}$(Ω, *λ*) is the surface emissivity as a function of direction and wavelength, and *I*_bb_ is the blackbody spectral radiance at a wavelength *λ* and temperature *T*_oc_.

**Fig. 4:**
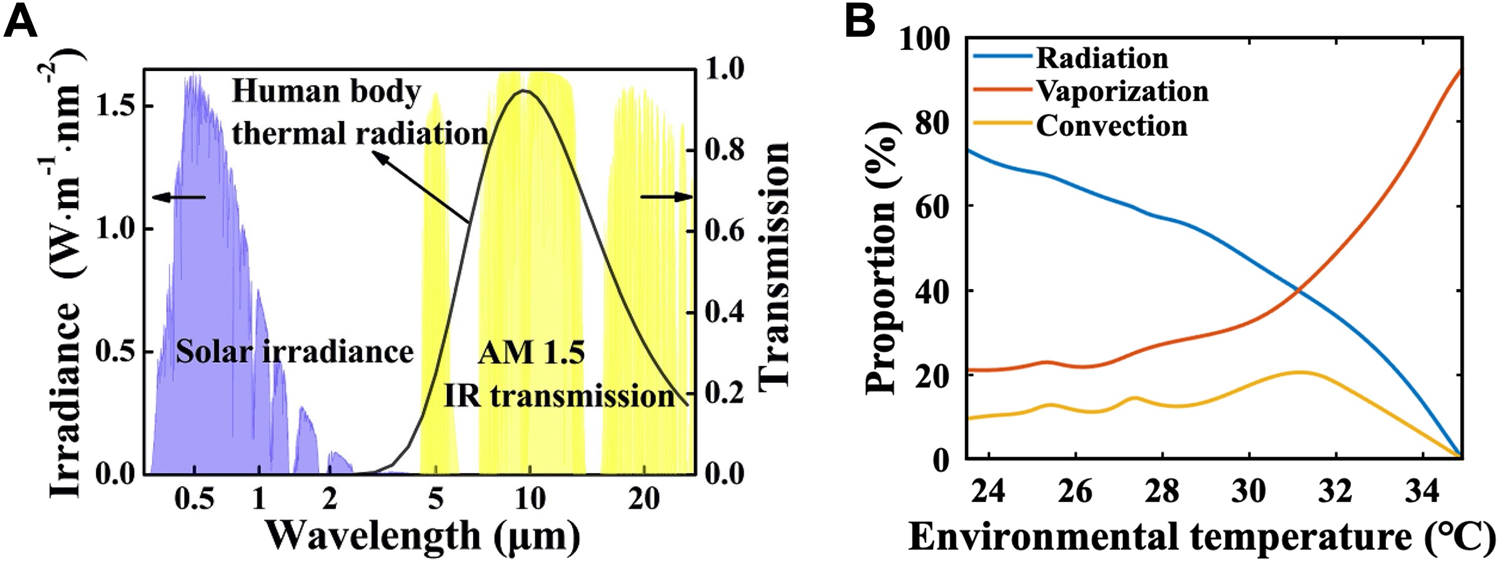
Illustration of the heat dissipation and thermal radiation spectrum of the human body. **a** Human body thermal radiation spectrum (solid dark line) calculated using the blackbody radiation law at a skin temperature of 34 °C. The AM 1.5G solar irradiance spectrum is shown as blue shading and the AM 1.5 atmospheric transmission spectrum in the IR region (4.2–26 μm) is shown as yellow shading [69]. Reproduced with permission. Copyright 2020, Elsevier. **b** The percentage of radiation, vaporization, and convection of human body heat losses as a function of environment temperature [12, 24]. Reproduced with permission. Copyright 2021, Elsevier

According to ISO EN 8996, the heat input generated from the normal metabolism is about 58 W m^−2^ for a seated and relaxed person and about 70 W m^−2^ for a seated person with sedentary activity like in a classroom or an office. For the standing person with slight activities, the metabolic heat generation is about 96 W m^−2^. Another significant heat input of the human body comes from solar radiation with a total power of about 1000 W m^−2^, and the solar spectrum is mainly distributed in the wavelength from 0.3 to 4 μm, as shown in Fig. 4a. More than 60% of the total solar irradiance can be absorbed by the human body skin [51].

In previous studies, the experimental findings have depicted the contributions of evaporation, convection, and radiation heat transfer to heat loss at different ambient temperatures [12, 24], as shown in Fig. 4b. It indicates that the thermal heat loss by radiation accounts for about 50% of the total heat loss of the human body in a typical indoor environment, demonstrating that regulation of radiation heat transfer has enormous advantages for personal thermal comfort regulation. Usually, the emissivity of human skin is approximately equal to that of a blackbody (about 0.98), making it an exceptional IR emitter. The radiative thermal energy from the human body is mainly distributed in the MIR wavelength range between 7 and 14 μm when the skin temperature is at 34 °C. And the peak of the human body emission wavelength is between 9 and 10 μm [51], as shown in Fig. 4a. The clothes that are considered as the second skin of the human body play a significant role in the heat transfer between the human body and the ambient environment. They act as a barrier for the inner microclimate against the environment. For example, they are desired to block heat input from the sunlight and allow heat dissipation from the human body when people are in hot environments. Conversely, the clothes are expected to block heat dissipation from the human body for keeping people warm in cold weather. Therefore, a suitable design of the cloth is of vital importance for efficient PTM.

### Regulation Principle of Radiative Heat Transfer

**Fig. 5:**
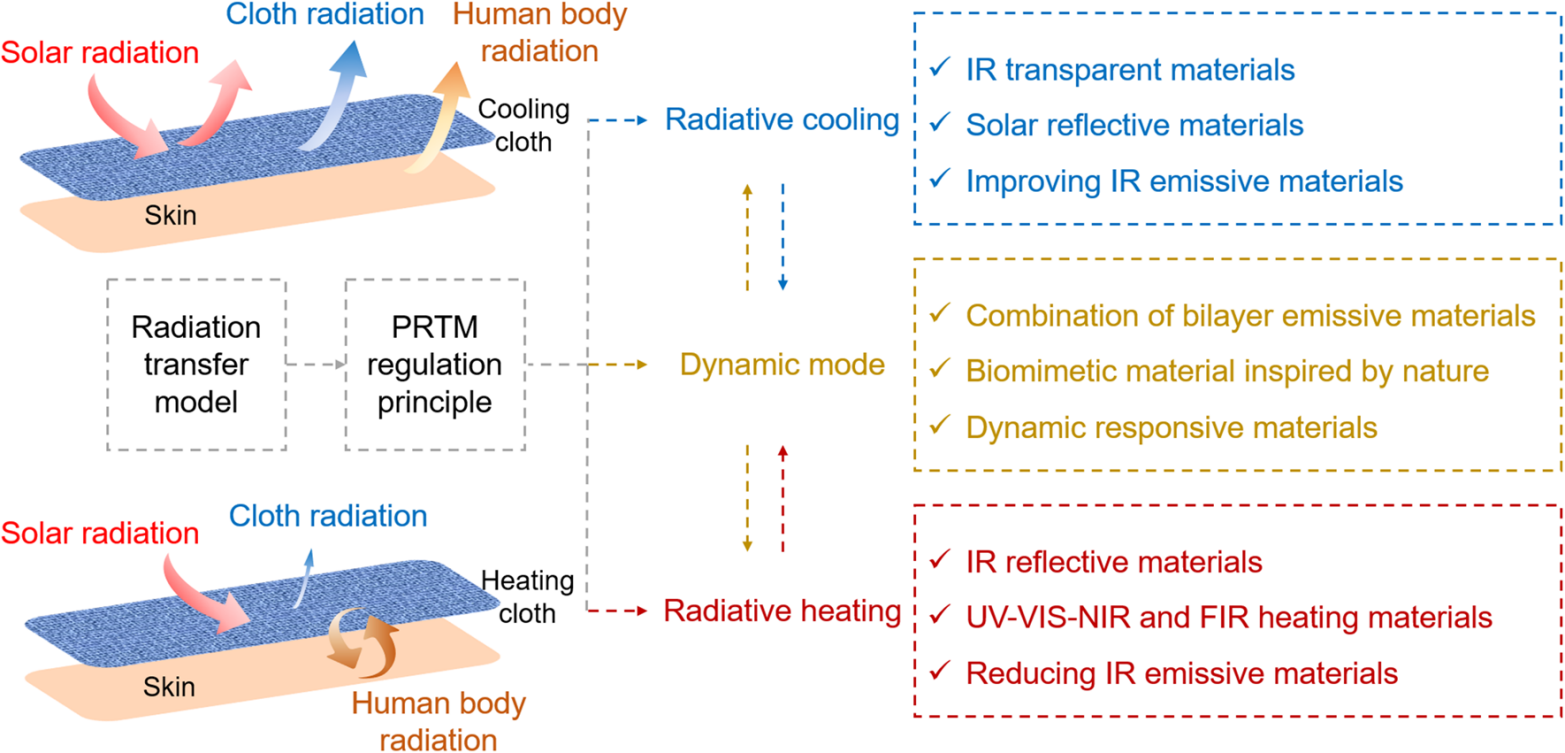
Schematic of the regulation principle of personal radiative thermal management

The application of radiative heat transfer for regulating the human body’s thermal comfort has promising potential due to its passive nature and high efficiency. Currently, most research efforts of passive radiative thermal management focus on surface modification or fabrication of textile materials to achieve the desired thermal comfort. Specifically, the optical properties of the fabric surface, such as the IR transmittance, reflectance, emissivity, and solar reflectance, are desired to be tailored or optimized in different directions for regulating the skin temperature of the human body. The regulation principles and structure design strategies are summarized in Fig. 5 and Table 2.

**Table 2:** Regulation principles and material/structure design strategies for PRTM

Functionality	Design principle	Material/structure design strategy
*Radiative cooling*		
IR transparent	Ideally, ${\tau }_{{\text{c}}}$ = 1 for IR radiation	Chemical bond stretching or bending vibration away from 7 to 14 μm, such as PE, PP, Nylon 6, PTFE, and PVDF. Smaller pore size for Rayleigh scattering of IR radiation
Solar reflective	Ideally, ${\rho }_{{\text{c}}}$ = 1 for solar radiation	Micro/nanoparticles with high refractive index, such as ceramic or inorganic nanoparticles, like TiO_2_, SiO_2_, and Al_2_O_3_
Improved IR emissive	Ideally, ${\varepsilon }_{{\text{c}}}$ = 1 for IR radiation	Metamaterials and multilayered nanophotonic structures with high emissivity at ATSW (8–13 μm)
*Radiative heating*		
IR reflective	Ideally, ${\rho }_{{\text{c}}}$ = 1 for IR radiation	Inclusion of metal-based materials with high IR reflectance, such as metal nanowires, metal nanoparticles, and metal composite, like as AgNWs, Ag NPs, and Cu–Ni NWs
Reduced IR emissive	Ideally, ${\varepsilon }_{{\text{c}}}$ = 0 for IR radiation	Nanophotonic structures coupled with metallic fibers with low IR emissivity, like nanoporous Ag and steel yarns
UV–VIS–NIR and FIR heating	Photothermal conversion for solar radiation absorption	Inclusion of high solar radiation absorptive materials, like CNTs, and dielectric layer, like Ge and ZrC
*Dynamic mode*		
Bilayer emitter	Combination of different emissive materials. High emissive layer faces outside and low emissive layer faces inside for cooling, and otherwise for heating	
Bionic materials	Composite materials with self-tunable thermoregulatory properties inspired by the structures of natural creatures, like chameleons and cephalopods	
Smart responsive materials	Inclusion of smart self-adaptive fibers that are responsive to environment temperature and humidity to change the yarn width or pore size	

In typical indoor scenarios, it is important to ensure effective dissipation of the heat generated by the human body’s normal metabolism into the surrounding environment. Therefore, the fabric designed should minimize any hindrances to the transmission of IR emitted by the human body. In other words, the emissivity of the designed cloth materials is ideally near one (${\varepsilon }_{{\text{c}}}$ = 1), or the fabric can be IR transparent (${\tau }_{{\text{c}}}$ = 1). The use of IR transparent fabric allows for direct dissipation of human radiation from the fabric surface, without the need for heat transfer from the skin to the fabric surface. However, when using an IR emissive fabric, the heat must first be transferred to the fabric surface before being dissipated through the outer surface. For the corresponding material selection and structure design, the chemical bond stretching and bending vibrations are required to be away from the human radiation wavelength for low IR absorption, as well as the pore size should be smaller than the IR wavelength for requiring Rayleigh scattering. Besides, the multilayered metamaterials with nanophotonic structures are efficient for improving the IR emission at atmospheric transparency spectral windows (ATSW), which will be discussed in Sect. 3.3. When people are exposed to the sunlight in outdoor environments, especially those who work outside for a long time like construction workers, the ideal textiles are also desired to reflect solar radiation or keep the sunlight opaque for blocking the heat input. Correspondingly, the solar reflectance of the outer fabric surface is supposed to be near one (${\rho }_{{\text{c}}}$ = 1 for solar radiation). Ceramic or inorganic nanoparticles with the high refractive index that could be regulated by the particle type and pore size are excellent solar reflective candidates.

In contrast to radiative cooling, the purpose of radiative heating or warming is to block the heat radiation loss from the human body as much as possible. As such, the functional textiles are desired to reflect the IR emitted from the human body on the inner cloth surface as well as reduce the IR emission on the outer cloth surface, suggesting that the IR reflectance of the inner surface is ideally equal to one (${\rho }_{{\text{c}}}$ = 1 for IR radiation), or the IR emissivity of the outer surface is as small as possible. These candidate materials are usually fabricated based on metals with low IR emissivity and high conductivity. Moreover, the metal-based materials can allow Joule heating for warmth sensation. In outdoor environments, it is more effective for warming up the human body if the fabric material can absorb the heat from solar energy, including ultraviolet light (UV), visible light (VIS), and near-infrared light (NIR). In addition, far-infrared light (FIR) with a longer wavelength can be absorbed by the epidermal layer of the human skin, leading to a thermal sensation of pleasant warmth. In other words, it requires solar absorptive materials for achieving body heating by photothermal conversion.

With the rapid advancements in material science and nanotechnology, numerous innovative dynamic fabric materials capable of achieving both heating and cooling modes for self-thermoregulation have been increasingly introduced. Dynamic textiles have the ability to adjust the thermal sensation from discomfort to comfort in various environments, while also enhancing the functionalities of clothing. As shown in Eq. (6), it demonstrates that the radiative heat transfer is mainly determined by the surface emissivity and temperature of the cloth. Therefore, it requires a rational combination of multilayer fabric materials with different emissivities to achieve heating and cooling based on the ambient environments. In other words, the higher emissive layer is designed to face outside in hot environments for dissipating the heat from the human body as much as possible. Conversely, the lower emissive layer should face outside when people are situated in cold environments for hindering heat loss. In the past few years, bionic materials have undergone rapid and booming development inspired by natural creatures, which shows considerable potential for thermal regulation fields and other traditional fields. Inspired by the natural creatures that can instinctively change their skin color to escape from danger, researchers have designed and fabricated dynamic textiles whose surface structures can change with ambient actuation for facilitating IR transmission or reflection. In addition, smart textiles can also be designed by incorporating the geometric changes of fibers or yarns that are responsive to ambient temperature or humidity. It was found that the yarn structures like yarn diameter and interspacing, as well as the fabric thickness, can cause significant effects on the optical properties of the fabric surface [70–73]. Therefore, the traditional fabric can be transformed into dynamic textiles with the bidirectional function of heating and cooling by rational design of responsive materials that are sensitive to ambient temperature, humidity, light, PH, or pressure.

### Evaluation of Personal Radiative Heat Transfer

**Fig. 6:**
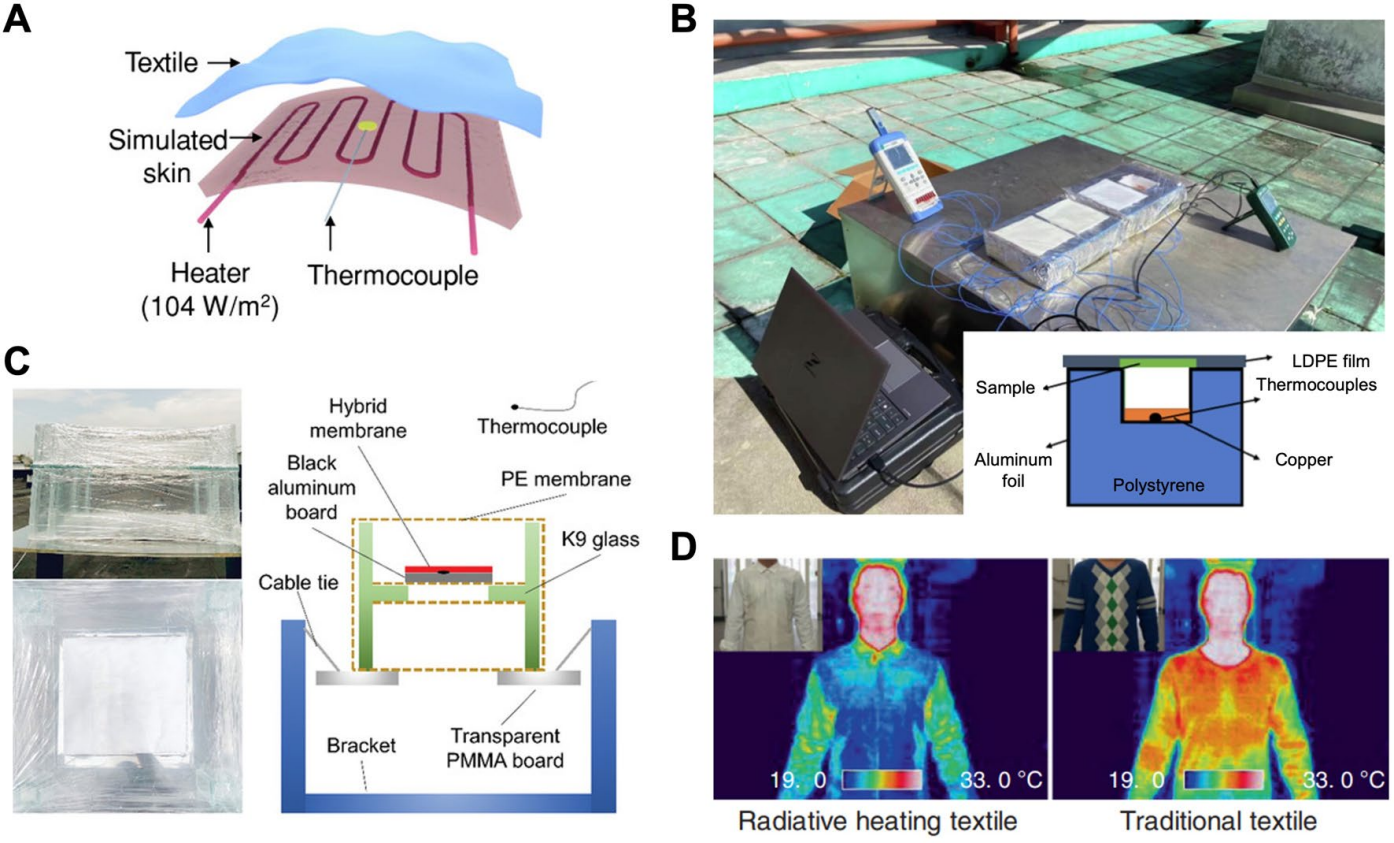
Evaluation methods for radiative heat transfer. **a** Schematic of the thermal performance measurement set-up of a simulated skin [51]. Reproduced with permission. Copyright 2018, Wiley-VCH Gmb. **b** Photograph of the outdoor experimental set-up enclosed by a thermal insulation foam and covered with a layer of aluminum foil and low-density polyethylene [75]. Reproduced with permission. Copyright 2022, Elsevier. **c** Digital pictures and illustration of the self-made, light-transmitting device [74]. Reproduced with permission. Copyright 2019 Wiley-VCH Gmb. **d** Thermal imaging and photographs (insets) of the human body wearing garments made from radiative heating textile and traditional textile for comparison [76]

Evaluation of the thermal performance of advanced textiles for PRTM, in past research, typically involves situating these textiles on either simulated skin or a human subject. The temperature distribution on the textile is generally determined using high-precision thermocouples and infrared thermal imaging devices. As shown in Fig. 6a, a constant heating power input is applied to the heater to simulate the metabolic heat generation rate of the skin. For outdoor thermal measurement, the sample is usually placed into a closed space, which is enclosed by a thermal insulation foam (Polystyrene) and covered with a layer of aluminum foil and low-density polyethylene (LDPE) for minimize convection and conduction heat loss, as shown in Fig. 6b. However, Wang et al. [74] discovered that the aluminum foil inevitably absorbed light and thus designed a light-transmitting device primarily composed of LDPE film (Fig. 6c). Highly transparent glass was used as a framework for stability, but sparingly to avoid potential interferences. On the other hand, when fabric samples are placed on an actual human body, the infrared thermal imager can more readily discern their thermal management performance. As shown in Fig. 6d, the human body heat was prevented from dissipating from the inner surface to the outer surface for the radiative heating textile, leading to a “cooler” outer surface compared to a traditional one. Although there have been various efforts toward evaluating radiative thermal performance, a standardized measurement protocol is currently lacking for both simulated skin and a Person’s actual body.

In the following sections, we will present and discuss advanced textile materials that have been recently proposed and designed. These materials provide novel functionalities for personal cooling, heating, and dual-mode applications. We will explore the design principles, fabrication methods, and heat transfer performances of these textiles, offering a comprehensive understanding of personalized thermal regulation materials.

## Advanced Textile Materials for Radiative Cooling

As discussed in Sect. 2.2, we will categorize personal radiative cooling materials into three types based on the radiative heat transfer models: IR transparent, solar reflective, and improved IR emissive materials. In the subsequent sections, these categories will be systematically examined and discussed, considering aspects such as fabrication methods, material structure, and thermal performance.

### IR Transparent Textile Materials

For personal cooling in hot weather, we hope to make textile IR transparent to fully dissipate human body radiation. An IR transparent textile would allow for cooling set-points to be higher while maintaining personal thermal comfort, with a 1–4 °C increase in temperature set-point translating to an energy savings of 7–45% [77]. “IR transparent” textile materials refer to those that can make IR radiation at 7–14 μm emitted from the human body through the textiles without obstruction. Tong et al. [70] first proposed a conceptual framework to thermally and optically design an infrared-transparent visible-opaque fabric (ITVOF) that achieves passive cooling by transmitting the thermal radiation directly to the ambience. It was numerically found that the fabric requires a minimum IR transmittance of 0.644 and a maximum IR reflectance of 0.2 to ensure thermal comfort at ambient temperatures of 26.1 °C. To meet the above requirements, an ITVOF are desired to be developed by structuring the composed fibers to minimize IR reflection via weak Rayleigh scattering while maintaining visible opaqueness via strong Mie scattering.

**Fig. 7:**
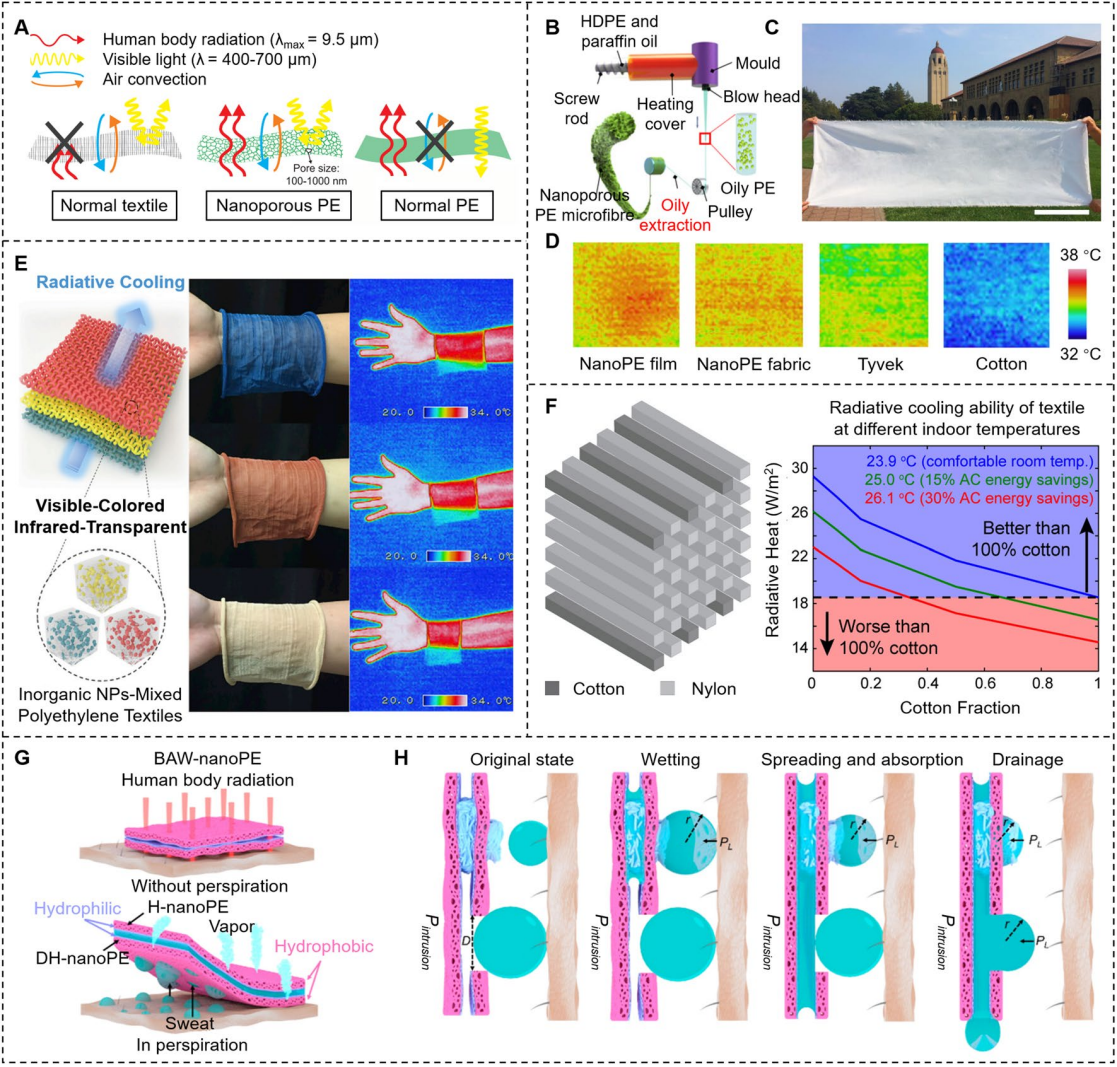
Representative IR transparent materials related to PE. **a** Schematics of comparison between nanoPE, normal PE, and cotton [71]. Reproduced with permission. Copyright 2016, American Association for the Advancement of Science. **b** A schematic diagram of the manufacturing process for the nanoPE microfiber. **c** A photograph of a large woven nanoPE fabric. Scale bar, 0.35 m. **d** Infrared images of the nanoPE fabric, nanoPE film, Tyvek, and cotton on simulated human skin [80]. Reproduced with permission. Copyright 2018, Springer Nature. **e** Photographs of colored knitted textiles and infrared images of bare skin and human skin covered with cotton for blue PB-PE, red Fe_2_O_3_-PE, and yellow Si-PE [81]. Reproduced with permission. Copyright 2019, Elsevier. **f** Radiative cooling ability of composite textile composed of a multilayered periodic array of parallel cotton and nylon fibers [83]. Reproduced with permission. Copyright 2016, American Chemical Society. **g** IR is transparent through bilayer anisotropic wettability nanoPE (BAW-nanoPE) without perspiration, and sweat can be drained in perspiration. **h** Water transportation mechanism scheme of fabricated BAW-nanoPE [85]. Reproduced with permission. Copyright 2012, American Chemical Society

The challenge for developing an ITVOF material is that the human body radiation spectrum (7–14 μm) overlaps with most of the IR absorption wavelength of common textile materials, such as C–O stretching (7.7–10 μm), C–N stretching (8.2–9.8 μm), aromatic C–H bending (7.8–14.5 μm), and S=O stretching (9.4–9.8 μm) [78, 79]. It indicates that most textile materials strongly absorb human body radiation and have very low IR transparency. Polyolefins such as polyethylene (PE) with only aliphatic C–C and C–H bonds were identified as potential candidate materials due to their intrinsically less absorptivity in the IR wavelength range, and the corresponding PE-based materials were widely designed and fabricated. Hsu et al. [71] presented a nanoporous polyethylene-based (nanoPE) textile that could promote effective radiative cooling while still possessing excellent air permeability, water-wicking rate, and mechanical strength for wearability after fabricated with the benign hydrophilic polydopamine (PDA). The sizes of interconnected pores were 50–1000 nm in diameter, which were comparable with the visible light wavelength (400–700 nm) and could make nanoPE opaque to human eyes by strong Mie scattering of visible light. Meanwhile, the pore sizes were much smaller than the IR wavelength, making nanoPE highly transparent to IR by Rayleigh scattering (Fig. 7a). The results showed that the skin temperatures were lower by 2.7 and 2.0 °C when covered with nanoPE cloth and with PDA-coated nanoPE cloth, respectively, than when covered with cotton.

Owing to the excellent IR transparency and VIS opacity of nanoPE, researchers have also extended various works from the perspectives of nanoPE microfiber forms, colored PE textiles with inorganic pigment nanoparticles, and the thermal ergonomics of nanoPE shirts [80–82]. To alleviate the discomfort associated with wearing fabric based on nanoPE film, such as the pronounced contact electrification and lack of tactile comfort, Peng et al. [80] developed a method for large-scale extrusion of uniform and continuous nanoPE microfibers. These fibers possess a cotton-like softness, making them suitable for industrial fabric production. This marked the first knitted and woven fabrics with nanoPE microfibers. Paraffin oil was selected as the solvent of PE and they could form a highly viscous homogeneous solution under a suitable temperature. After extruded by an industrial extruder machine, the solid-phase PE was separated from the liquid-phase paraffin oil to form nanoPE microfibers (Fig. 7b, c). The fabric containing nanoPE microfibers exhibited a great cooling power, lowering the human skin temperature by 2.3 °C than commercial cotton fabric (Fig. 7d), which corresponds to a greater than 20% saving on indoor cooling energy. Also, the nanoPE fabric has impressive wearability and durability. However, the IR transmittance was considerably related to the fabric thickness and the thicker fabric usually leads to a lower IR transmittance. While nanoPE-based textiles have been verified with a high IR transparent property, they still lack tunability in visible color. The commonly used organic dye molecules strongly absorb the IR radiation emitted from the human body, thereby lowering IR transparency and increasing IR emissivity. In addition, PE is chemically inert and lacks polar groups, inhibiting the surface adhesion of chemical dyes. Therefore, Cai et al. [81] proposed a strategy utilizing unique inorganic pigment nanoparticles, such as Prussian blue (PB), iron oxide (Fe_2_O_3_), and silicon (Si), as coloring components to achieve the coloration of IR transparent PE textiles for radiative cooling (Fig. 7e). The colored textiles could reflect certain visible colors through optimized concentration and size of pigment nanoparticles. The knitted fabrics showed a high IR transparency of about 80% and good radiative cooling performance of 1.6–1.8 °C, as well as good color stability against more than 100 washing cycles.

Inspired by the exceptional IR transparency of PE-based textiles, there has been an increased focus on enhancing the IR transmittance of traditional fiber materials. For instance, cotton fiber, which has a higher IR absorptivity due to the overlap of its molecular bond vibration range with the IR wavelength emitted from the human body, is receiving considerable attention. The development of composite textiles by blending IR transparent PE-based materials with other traditional materials is an effective approach to achieving better wearability and mechanical strength. Also, the optical properties of traditional fiber materials could be tailored by the design of photonic structures. For instance, Catrysse et al. [83] showed photonic structure textiles based on the blending of largely IR transparent fibers for efficient cooling and natural IR opaque fibers for wearing comfort (Fig. 7f). The numerical results suggested that the combination of up to one-third cotton and two-thirds nylon could allow net heat transfer at an extended temperature set-point of 26.1 °C that exceeded the cooling ability of a cotton-only design at the current thermal comfort set-point of 23.9 °C, which resulted in more than 30% energy savings. Furthermore, it is enlightening to note that conventional fabric materials like flax, silk, and polyester fibers could be combined with highly IR transparent textiles such as PE, polyamide (PA)/polycaprolactam (commonly known as Nylon 6), and polyvinylidene fluoride (PVDF), which could simultaneously achieve efficient cooling and optimal wearability [84].

While substantial progress has demonstrated that nanoPE can achieve superior thermal management due to its high IR transparency, research into nanoPE’s moisture management capabilities is still in its early stages, primarily due to its inherent hydrophobicity. Besides, when the cloth becomes wet due to untimely sweat drainage, the radiative cooling performance will reduce and people will undergo a sticky feeling. In order to realize moisture transportation and radiative cooling simultaneously, Hu et al. [85] designed a bilayer nanoPE membrane (BAW-nanoPE) with asymmetric microtopology and anisotropic wettability (Fig. 7g, h), which could realize rapid sweat drainage and good evaporation/radiative cooling performance as well as block permeation of water from the outside.

### Solar Reflective Textile Materials

Regulation of radiative heat transfer from the human body for cooling is sufficient under typical indoor sceneries without other intense radiation sources. But it is required to avoid solar thermal radiation with strong intensity in outdoor environments. The solar spectrum mainly consists of visible light at 400–700 nm and near-infrared light at 700–2500 nm, accounting for more than 90% of the whole solar irradiance (about 1000 W m^−2^). Therefore, developing advanced reflective materials to cut off the radiation energy from the sun is another decent strategy for personal cooling, especially when people are exposed to intensive sunlight. Previous studies have presented various types of solar reflective materials, including inorganic/organic pigments (e.g., chlorophyll [86], black pigments containing copper phthalocyanine [87], AZO pigments [88, 89], and a few perylene-based pigments [90]), and metallic based pigments [91–93].

The incident photons can be reflected by free electrons in metals, resulting in a high reflection of visible (VIS) and near-infrared (NIR) light on the surface. It has been proven that aluminized fabrics are among the most effective methods for reducing radiation heat flux and enhancing thermal insulation performance against solar radiation [94]. For example, Zhu et al. prepared the aluminum-foiled aramid fabric by a simple coating method and it exhibited a comparatively high average reflectance of more than 0.7 in the radiant wavelength of 1547–2500 nm [95]. Transparent conductive oxides are considered as one of the potential candidates for high infrared reflective materials due to their high concentration of free electrons. Miao et al. [96] modified the Polyethylene terephthalate-based (PET) fabric with AZO/Ag/AZO multilayer ceramic films by radio frequency magnetron sputtering for improving the solar reflectivity, which makes them potential candidate materials for solar control applications. The results indicated the thickness of the Ag layer had significant effects on the properties of coated films. It showed that the sample with a 15 nm thickness Ag inner layer had the highest infrared reflection rate of 96%. However, the aluminized fabrics are quite laborious in application due to their oxidation, flammability, delamination, and poor permeability.

**Fig. 8:**
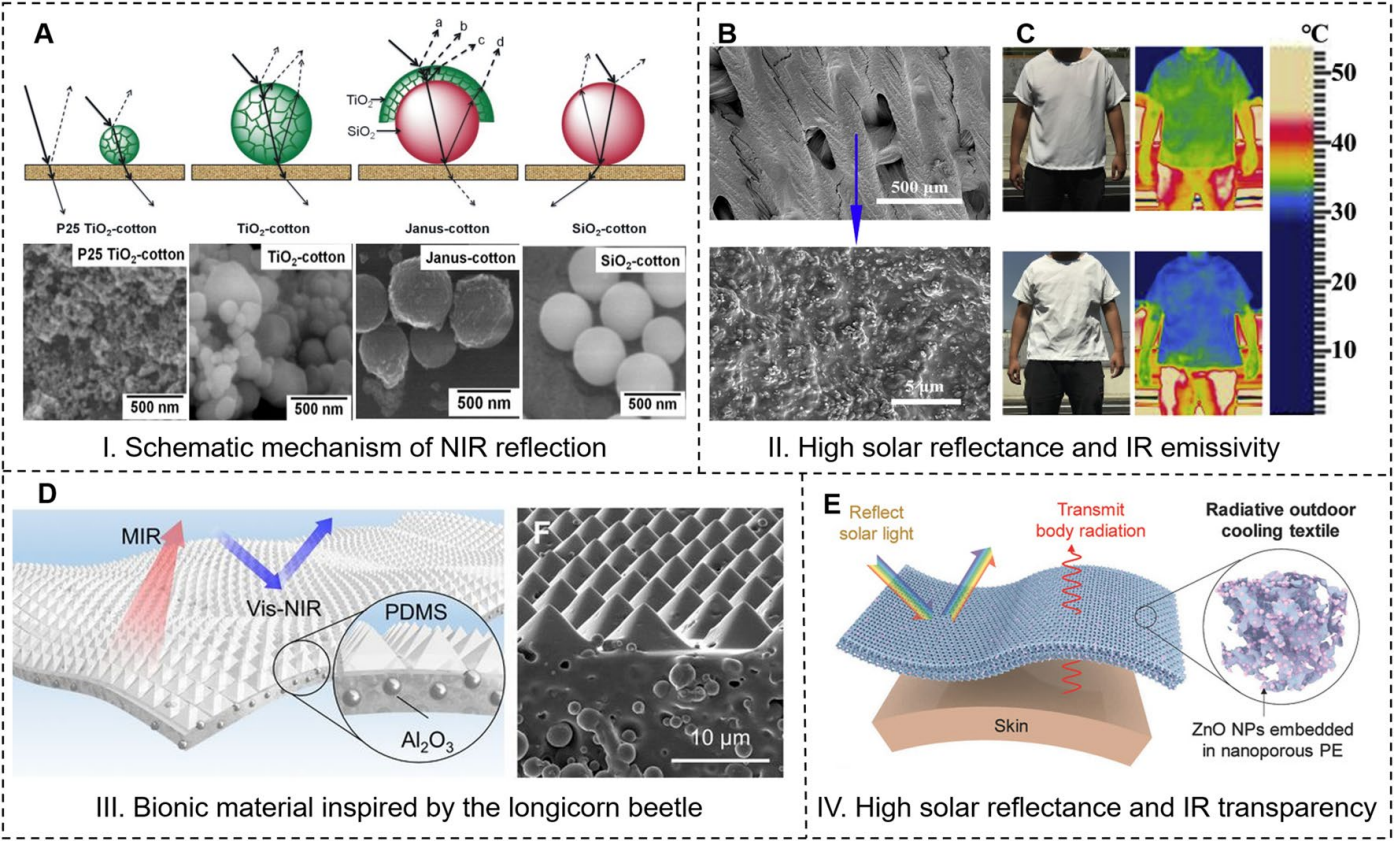
Representative solar reflective materials. **a** Schematic representation and SEM micrographs of the proposed mechanism of NIR reflection of P25 TiO_2_-cotton, TiO_2_-cotton, SiO_2_-cotton, and Janus-cotton [99]. Reproduced with permission. Copyright 2017, Elsevier. **b** SEM image of the top surface for Al_2_O_3_-cellulose acetate coated on Mitsubishi cellulose acetate textile showing the Al_2_O_3_ NPs embedded in cellulose acetate of modified textile [69]. **c** Infrared images of a Mitsubishi T-shirt (top), and a modified Mitsubishi T-shirt (bottom) on a clear summer day in Shanghai, China [69]. Reproduced with permission. Copyright 2020, Elsevier. **d** Schematic illustration of the bioinspired flexible hybrid films. Ceramic particles are embedded in the PDMS matrix filled by compact arrays of micropyramids [100]. **e** Schematic of the ZnO nanoparticle-embedded nanoporous PE textile, designed for radiative outdoor cooling by reflecting sunlight and transmitting human body thermal radiation [51]. Reproduced with permission. Copyright 2018, Wiley-VCH Gmb

Applying functional solar reflective coatings onto the surface of the fabric is a commonly employed technique to reflect solar radiation and improve thermal insulation. These coatings are usually composed of pigments and inorganic particles that can effectively reflect solar radiation. The main principle is to adopt the high refractive index of micro/nanoparticles to reflect NIR light, thereby blocking the fabric from absorbing solar radiation. And the type and size of embodied particles are considered as the main factors contributing to the sunlight reflection characteristic [97]. Wong et al. [98] obtained a cotton-based fabric coated with irregular-shaped TiO_2_ particles in diameter of 293–618 nm by calcination treatments and phase transition from anatase to rutile. It was found that the refractive index and particle diameter had a combined effect on the NIR reflectance of the TiO_2_ powder, and the highest solar reflectance of 84.80% occurred in the TiO_2_ sample with anatase: rutile ratio of 35:65 and a particle diameter of 563 nm. A lower surface temperature was recorded with a maximum difference of 3.9 ℃ for the calcined TiO_2_-coated cotton fabric. Besides, a chitosan-TiO_2_ coating could provide a better wash fastness than TiO_2_ alone. Also, Panwar et al. [99] explored the thermal regulation of TiO_2_-SiO_2_ Janus particles treated cotton fabric and showed a significantly high NIR reflectance of 79% and better thermal regulation than control cotton, P25 TiO_2_, TiO_2_, and SiO_2_ treated cotton fabrics. The higher reflectance was attributed to the multi-interface reflection, including the air-SiO_2_ interface, TiO_2_ crystal interface, TiO_2_–SiO_2_ interface, and SiO_2_-fiber interface (Fig. 8a).

In addition to the development of solar reflective material-coated textiles, considerable efforts have been dedicated to composite textile materials that possess both high solar reflectance and high IR emissivity. These advancements aim to achieve more effective thermal management. Wei et al. [69] modified a microstructured textile (Al_2_O_3_-cellulose acetate) by embedding aluminum oxide nanoparticles (Al_2_O_3_ NPs) into nanoporous cellulose acetate (CA) (Fig. 8b). Cellulose acetate presented a high emissivity in the long-wave of IR region which could promote effective thermal radiation (2.5–25 μm), while Al_2_O_3_ possessed high thermal conductivity and could enhance effective solar reflection. The solar reflectance of the original textile increased from 62.6 to 80.1% after modification, which reduced the temperature of simulated skin by 2.3–8 °C compared to that without modification. And the modified T-shirt avoided the overheating of actual human skin by 0.6–1.0 °C in a real-life cooling experiment, corresponding to a temperature decrease of 1.9–3.3 °C for the internal surface of the textile. Natural evolution has resulted in organisms with excellent thermoregulation capabilities, especially in extreme climates. The development of bioinspired materials that mimic biological properties for thermoregulation has proven promising for passive radiative cooling. Inspired by the longicorn beetle’s excellent thermoregulation function with their triangular cross-section fluffs that effectively reflect sunlight and emit thermal radiation for body temperature control, Zhang et al. [100] designed and demonstrated a photonic material consisting of a periodic micro-pyramid-arrayed polydimethylsiloxane (PDMS) matrix encapsulating randomly distributed spherical ceramic particles (Al_2_O_3_) (Fig. 8c). The film presented a solar irradiance reflectance of about 95% and an IR emissivity of more than 96%. Under direct sunlight, it showed a cooling power of about 90.8 W m^−2^ and a temperature decrease of up to 5.1 °C. Generally, the pores or voids inside fiber were often regarded as the scattering center for solar radiation reflection when the cross-section sizes were comparable to the wavelengths of VIS and NIR lights. The natural silk cocoon fibers are populated with a high density of air voids randomly distributed across the fiber cross-section but are invariant along the fiber, which can strongly scatter the sunlight. A silk fiber with a thickness of 50 μm can reflect 66% of solar radiation while showing a high MIR emissivity of 88%, making it an efficient radiative cooling device. Drawing inspiration from the above fiber, Shi et al. [101] fabricated biomimetic nanostructured fibers based on both regenerated silk fibroin and PVDF by wet spinning. The optical characterization showed that nanostructured regenerated silk fibers presented a solar reflectance of 73% and a thermal emissivity of 90%, and nanostructured PVDF fibers provided a solar reflectance of 93% and a thermal emissivity of 91%. It was found that the filamentary air voids led to highly directional scattering and gave the fibers a highly reflective sheen. Similarly, Wang et al. [74] fabricated a high-performance flexible hybrid membrane radiator (FHMR) whose inside contained numerous nanoporous PVDF/tetraethyl orthosilicate (TEOS) fibers and the outer surface consisted of random SiO_2_ microspheres. A 300-µm-thick FHMR showed an average IR emissivity of more than 96% and solar reflectance of about 97%, as well as great flexibility and superior strength. An average radiative cooling power of 61 W m^−2^ and a temperature decrease up to 6 °C under a peak solar intensity of 1000 W m^−2^ were achieved.

It would be more efficient for personal cooling, particularly in outdoor situations, to manipulate both solar radiation and human body radiation simultaneously. In other words, the development of advanced textile materials capable of reflecting visible (VIS) and near-infrared (NIR) lights, as well as allowing the IR emitted from the human body to pass through transparently, is necessary. To reduce solar radiation while facilitating human radiation for outdoor cooling, as shown in Fig. 8d, Cai et al. [51] designed a novel spectrally selective nanocomposite textile using zinc oxide nanoparticle-embedded polyethylene (ZnO-PE) through the combination of intrinsic material properties and structure photonic engineering, which could reflect more than 90% of solar irradiance and enable simulated skin to avoid overheating by 5–13 °C compared to cotton under peak daylight condition.

### Improved IR Emissive Textile Materials

In addition to developing materials that are transparent to IR radiation for radiative cooling, enhancing the IR emissivity of textile surfaces is another effective approach to realize thermal comfort regulation. The atmospheric transparency spectral windows are a wavelength range of 8–13 μm for electromagnetic radiation and allow infrared energy to get through (Fig. 4a), leading to radiative cooling of the surface directly oriented to outer space, which is also called “daytime radiative cooling” [65, 102, 103]. The wavelength peak of thermal radiation at a typical atmospheric temperature on the earth’s surface coincides with the aforementioned atmospheric transparency windows, meaning that the object on earth could dissipate the heat into outer space by electromagnetic waves in the 8–13 μm. Methods that harness nanophotonic structures have successfully yielded daytime radiative cooling systems, however, most current radiative cooling structures, such as thin films, coating, and paints, still result in weak air–water permeability and inadequate wearability that limit the direct applications to PRTM [18, 104, 105].

**Fig. 9:**
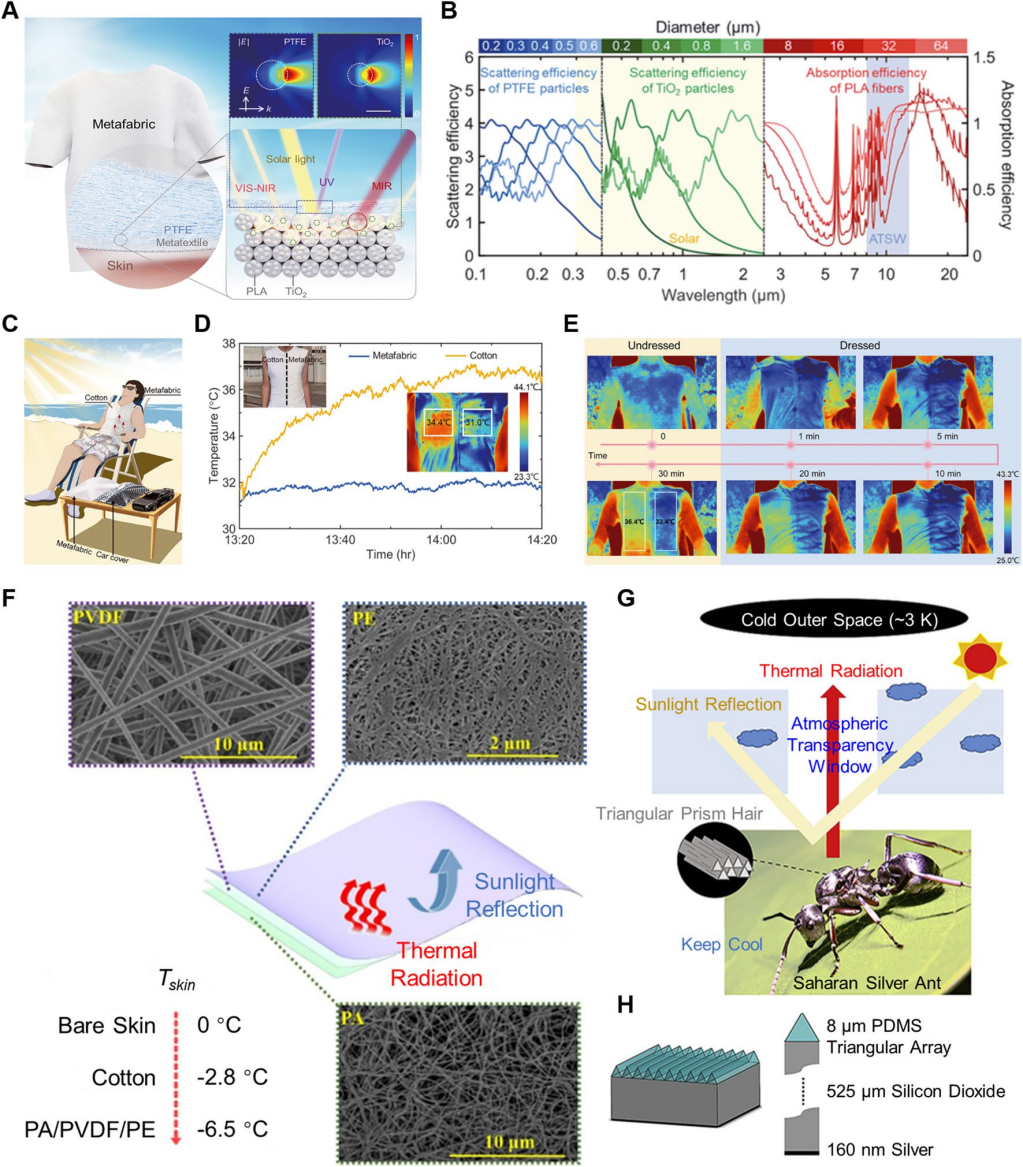
Designs of metafabric material, nature-inspired multilayer nanophotonic structures, and bionic structures for radiative cooling. **a** Schematic of a metafabric for daytime radiative cooling. The blue, green, and red dashed boxes highlight the three-level hierarchical structure responding to the UV, VIS–NIR, and MIR bands, respectively. The insets show the calculated scattering fields of 300- and 550-nm light by a 500-nm PTFE particle and a 400-nm TiO_2_ particle, respectively. Scale bar, 400 nm. *E*, the electric field of the incident light; *k*, the wave vector of the incident light. **b** Calculated scattering and absorption efficiencies for particles with different sizes encapsulated in the metafabric. PTFE particles, TiO_2_ particles, and PLA fibers demonstrate strong scattering and absorption of UV light, VIS–NIR light, and MIR light, respectively. **c** Schematic of the metafabric cooling tests on the human body. **d** Temperature tracking for skin under different fabrics in direct sunlight in Guangzhou, China. **e** Infrared images of the volunteer under direct sunlight in Sipsongpanna, China [18]. Reproduced with permission. Copyright 2021, American Association for the Advancement of Science **f** Microstructure of the PA/PVDF/PE composite textile [107]. Reproduced with permission. Copyright 2020, Elsevier. **g** A schematic diagram of thermoregulatory effect discovered in the Saharan silver ant, Cataglyphis bombycina. **h** The pattern of designed PDMS-SiO_2_-Ag with a triangular prism array of a size parameter at 8 μm inspired by the Saharan silver ant [111]. Reproduced with permission. Copyright 2020, Elsevier

Numerous studies have concentrated on advancing different radiative cooling metamaterials with unique optical properties. These include a high solar reflectance to block radiation input and a high IR emissivity to facilitate radiation dissipation. Moreover, the focus has been on achieving flexible wearability in these materials. For example, Zeng et al. [18] designed a multilayer metafabric knitted with composite microfibers, which incorporated hierarchically designed metamaterial structures to directly integrate radiative cooling technology (Fig. 9a). The bottom layer consisted of titanium oxide-polylactic acid (TiO_2_-PLA) composite woven textile, which embodies nanobeads of 200–1000 nm in diameter and nanofibers with a length of several micrometers and a width less than 200 nm. The laminated top layer is a 50-μm-thick polytetrafluoroethylene (PTFE). PTFE particles strongly reflected UV light from the incoming radiation, and TiO_2_ nanoparticles with high refractive indices produced the scattering peaks required to cover the entire VIS–NIR band with high efficiency. PLA microfibers could provide rich emittance in the MIR wave band, high moisture absorption property, and substantial biodegradability (Fig. 9b). The hierarchical-morphology design enabled the metafabric to resonantly reject solar power and strongly emit in the MIR range. The fabric showed a broadband reflectance of 92.4% in the solar radiation region (0.3–2.5 μm) and an average emissivity of 94.5% across the ATSW. The outdoor experiment under direct sunlight in Guangzhou showed a large temperature difference between the two sides of the vest (34.4 °C with cotton and 31.0 °C with metafabric) (Fig. 9c, d). A similar test performed in Sipsongpanna suggested that the two halves of the body showed a distinct temperature difference of more than 3 °C directly after the vest was removed (Fig. 9e).

Many multilayered nanophotonic devices that could reflect the solar irradiance and emit strongly in the infrared band have also been proposed and applied for radiative cooling [67, 106]. As shown in Fig. 9f, a tri-layered structured composite textile made from PA, PVDF, and PE was designed for radiative cooling in indoor and outdoor environments [107]. The PA/PVDF/PE composite textile could strongly absorb/emit thermal radiation at the atmospheric window (8–13 μm) while minimizing the absorption/emission in other infrared wavelengths (2.5–8 and 13–25 μm). Besides, it could reflect more than 90% of the visible light (300–800 nm) due to the inside micro-nanopores. It could decrease the body temperature by 6.5 °C under direct sunlight with a high cooling capability, about 2.5 times that of traditional natural textiles. Furthermore, Song et al. [108] proposed fabricated radiative cooling fibers by utilizing PE and polyethylene oxide (PEO) as raw materials. The porous PE fiber with pore and wrinkle structures endowed a high IR emissivity of 90.97% in 8–13 µm and a sunlight reflectance of 93.77%. The outdoor cooling measurement under direct solar irradiation showed that the porous PE textile avoided the human body and the transparent house overheating by 6.8 and 20.3 °C, respectively. Gu et al. [109] modified a laminated membrane that combined zinc-aluminum layered double hydroxides /cotton fiber (CF@Zn–Al LDHs) and zinc oxide nanorods/cotton fiber (CF@ZNR) via a vacuum filter pump. The inner layer of CF@Zn–Al LDHs exhibited a maximum IR emissivity of 98% and low reflectance of 1.0% to achieve infrared dissipation, while the CF@ZNR layer possessed a high IR transmittance of about 83.0%.

In addition, the natural animals in extremely hot environments can motivate researchers to artificially design and fabricate hierarchical structures. Saharan silver ants can maintain their body temperature due to unique triangular-shaped hairs that enhance solar reflection and thermal emission through ATSW (Fig. 9g) [110]. Inspired by it, Jeong et al. [111] presented a geometrically modified polymer-based daytime passive radiative cooler, which was composed of a MIR emissive upper layer with PDMS and SiO_2_ and the solar reflective bottom layer containing Ag (Fig. 9h). The average emissivity in the 8–13 μm spectrum was enhanced to 98% by the gradient refractive index effect, while the average solar reflectance in the VIS and NIR spectrum was measured to be 95%. Also, it was numerically found that the prismatic structure on the top surface of SiO_2_ could enhance the MIR emittance. With the hot and humid climate in Hong Kong, a field test successfully demonstrated cooling by 6.2 °C below the ambient temperature.

## Advanced Textile Materials for Radiative Heating

As outlined in Sect. 2.2, the materials required for personal radiative heating primarily fall into three categories: IR reflective, reduced IR emissive, and solar absorptive. These categories will be organized and discussed in a manner similar to the radiative cooling materials, as presented from Sects. 3.1 to 3.3.

### IR Reflective Textile Materials

**Fig. 10:**
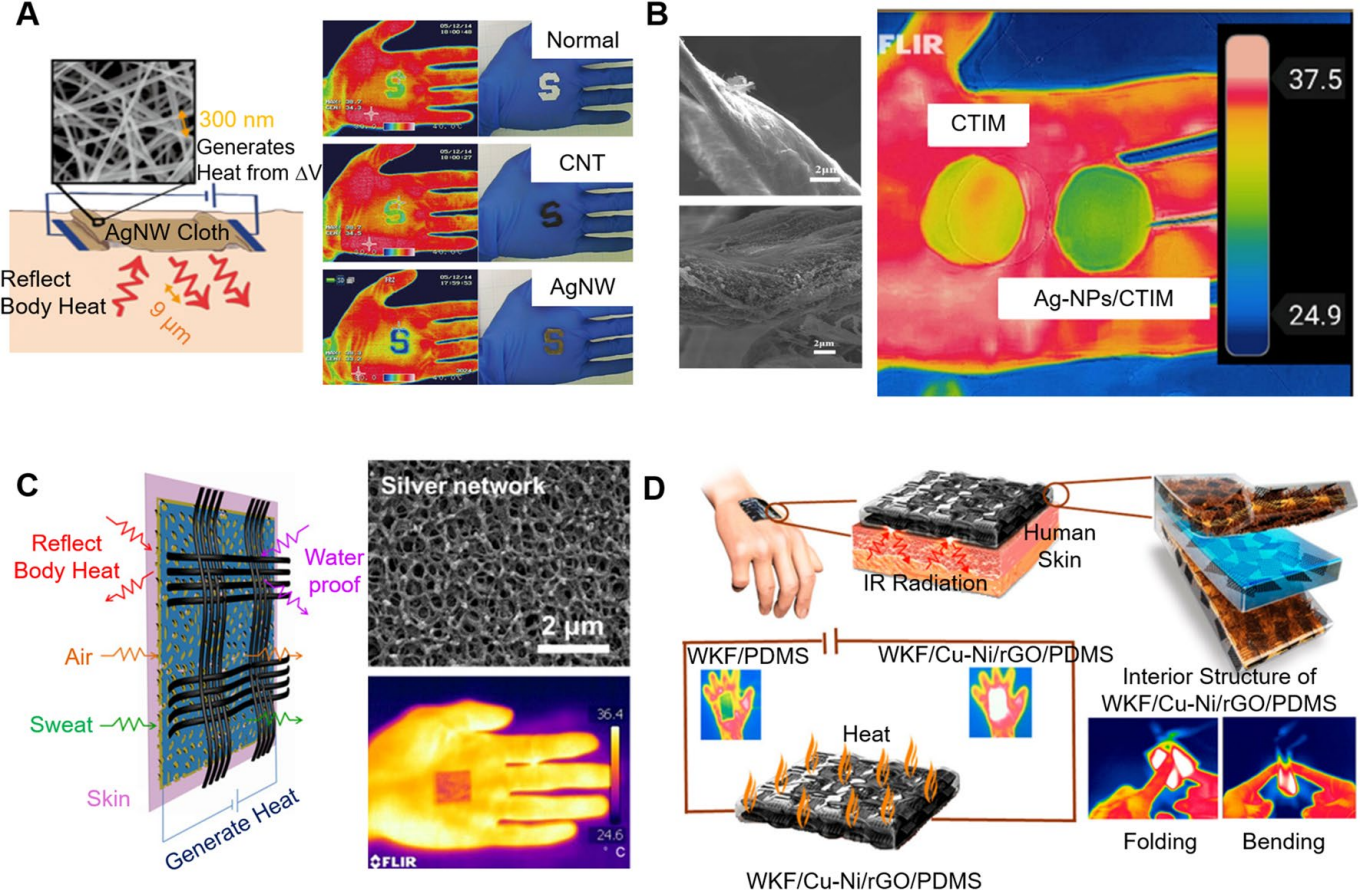
Metal-based IR reflective materials. **a** Schematic of nanowire cloth with thermal radiation insulation and active heating, and the corresponding thermal images and regular photographs of normal cloth, CNT-cloth, and AgNW-cloth [114]. Reproduced with permission. Copyright 2015, American Chemical Society. **b** SEM images and thermal images of the CTIM and Ag NPs/CTIM [116]. Reproduced with permission. Copyright 2019, Elsevier. **c** Schematic of multifunctional cotton-based cloth modified with a superhydrophobic silica nanoparticle/PDMS layer on one side and coated with a nanoporous cellulose acetate layer followed by depositing a thin silver film on the side. Also, it showed the SEM image and infrared photograph [112]. Reproduced with permission. Copyright 2020, American Chemical Society. **d** Schematic of WKF/Cu-Ni/rGO/PDMS composite [117]. Reproduced with permission. Copyright 2020, American Chemical Society

Compared to cooling, much larger energy savings can be significantly expected by developing radiative heating textiles because space heating (about 22.5%) accounts for a much larger proportion of all the energy consumed in the building sector than space cooling (about 14.8%) [76]. For radiative heating or warming of the human body, the most widely investigated advanced textile materials are those with high IR reflectance. It was found that such functional textiles were mostly constructed by the fabrication or inclusion of metal-based materials via physical and chemical deposition methods. Metals were considered as a type of good IR reflector and have been extensively used in the life of people [112]. However, metal foils or membranes are not suitable for human insulation materials due to their heavy weight, weak breathability, and discomfort wearability. By contrast, metal nanowires have attracted more attention because of their high IR reflectance and the network structure formed by metal nanowires, improving the breathability of metal-based composite materials [113]. Among them, silver is a prior candidate due to its high IR reflection efficiency, excellent resistance to acid and alkali, as well as stable antioxidation ability. Hsu et al. [114] demonstrated a silver nanowire-embedded cotton (AgNWs) cloth by dip-coating method. The metallic nanowires formed a conductive network that not only highly reflected IR radiation from the human body but also allowed Joule heating to complement the passive insulation (Fig. 10a). Thermal images indicated that the measured temperature was at 30–31 °C for the AgNWs-coated cloth, while was 33–34 °C for the normal cloth and CNT-coated cloth. The high reflectance of the AgNWs coating made AgNWs-coated cloth “colder” than other clothes as shown in thermal images. Besides, the breathability and durability of the AgNWs-coated cotton cloth were not sacrificed due to the formed porous structure. Also, Yu et al. [115] modified a PTM cotton-based cloth using a coating of AgNWs and PDA nanocomposite by intermolecular cross-linking. The AgNWs/PDA cloth highly reflected MIR to FIR radiation from the human body with an average reflectance of 86%, about 66 times higher than the normal cloth. Meanwhile, it allowed Joule heating with a quick thermal response, increasing from 22 to 40 °C in 1 min. Additionally, the cloth was durable and washable. Despite these advantages, the real applications of AgNWs were hindered by the extremely expensive cost and the complicated preparation process. Later, a multifunctional Ag nanoparticles/cellulose fibers membrane was prepared by a simple silver mirror reaction and vacuum filtration to improve the IR reflection for human thermal insulation [116] (Fig. 10b). The membrane exhibited high IR reflection efficiency, good breathability for wearing comfort, and excellent antibacterial property.

Besides the surface modification method discussed above, the combination of multilayer materials with different functions can also achieve the heating or warming of the human body. Liu et al. [112] designed a multifunctional cotton-based fabric, whose one side was a superhydrophobic layer consisting of silica nanoparticles and PDMS, and the other side was coated with a nanoporous cellulose acetate layer followed by depositing a thin silver film (Fig. 10c). The porosity allowed the fabric to be breathable, and the silver film acted as a perfect infrared reflector, a flexible heater, and an antibacterial layer. The average transmittance in the range of 2.5–16 μm for the multifunctional cloth was close to 0%, resulting in a decreased surface temperature of 1.3–1.6 ℃ on infrared images compared with that of the normal cloth. A woven Kevlar fiber (WKF) fabricated by copper-nickel nanowires (Cu-Ni NWs) was combined with reduced graphene oxide (rGO) dispersed in PDMS to form composites using vacuum-assisted resin transfer molding [117] (Fig. 10d). The Cu–Ni NWs and rGO could promote effective Joule heating and reflect the IR radiation emitted by the human body. It showed that the average IR reflectances of all the composites that contained Cu–Ni NWs and rGO were more than 98%, and the Cu_3_Ni_1_-WKF/PDMS provided 43% more thermal insulation than bare WKF/PDMS with an IR reflectance of 84.2%. MXene, as a new two-dimensional material with remarkable properties, has been widely applied in wearable electronics [118]. Hazarika et al. [119] reported a self-powered woven WKF-based flexible PTM device with a porous Ag@Mo_*x*_Fe_1−*x*_Se nanostructure between WKF and Ti_3_C_2_ MXene film dispersed in PDMS, showing a MIR reflectance of 97.4% and a UV–VIS–NIR absorptivity of 87.10%. Besides, the fabricated device could harvest energy from the human body movements, exhibiting high self-powered heating efficiency with a maximum power density of 1.5 mW cm^−2^ at 5 Hz.

### Reduced IR Emissive Textile Materials

**Fig. 11:**
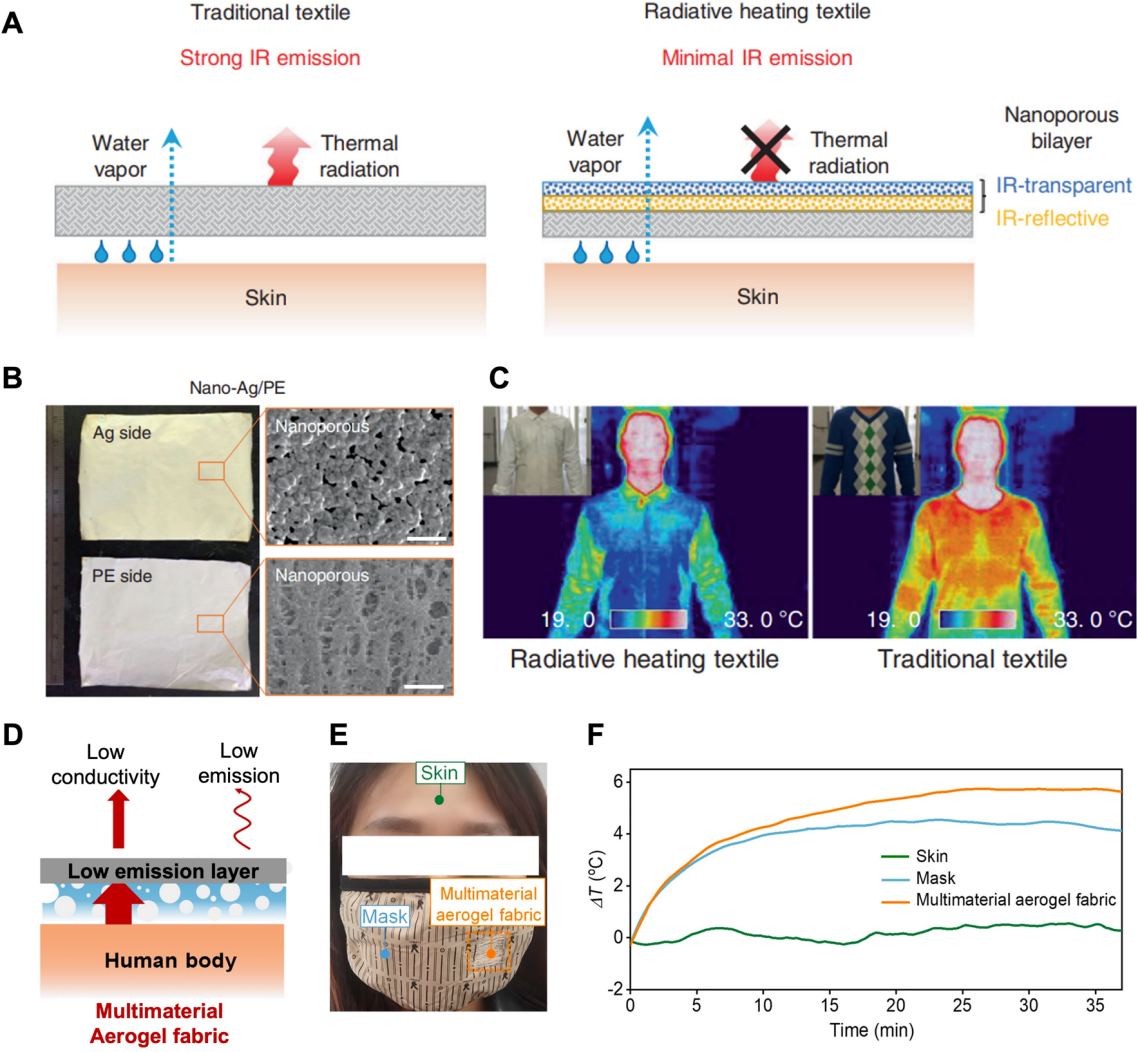
**a** Schematics depicting the heat dissipation and vapor transmission of the human body covered with traditional textile and nanophotonic structure heating textile composed of an IR transparent layer and an IR reflective layer with embedded nanopores in both layers to simultaneously achieve minimal IR emissivity and good breathability. **b** Photographs and SEM images of the Ag side and PE side of nano-Ag/PE. Scale bar, 1 µm. **c** Thermal imaging and photographs (insets) of the human body wearing garments made from radiative heating cotton/Ag/PE textile and traditional textile, respectively [76]. **d** Schematic of the heat dissipation of the human body with multi-material aerogel fabric. **e** Digital photograph of commercial cotton mask integrated with a piece of multi-material aerogel fabric by weaving (orange dashed box). The colored points are the positions of the thermocouples.** f** Real-time temperature difference ∆*T* between multi-material aerogel fabric, mask, and bare skin [120]. Reproduced with permission. Copyright 2022, Springer Nature

The existing radiative heating textiles are mostly based on the concept of reflecting the human body IR radiation to reduce heat dissipation. However, there always exists a dilemma between optimal heat transfer performance and excellent wearability, which could be addressed by designing the advanced photonic structure. Besides, it is more effective for the thermal insulation performance of the fabric if the IR radiation emitted from the human body is blocked while the thermal radiation loss from the outer surface of cloth is also suppressed. Interestingly, it was found that the outer textile surface IR emissivity plays a decisive role in the textile’s heating performance. Heat conduction is dominant over radiation between the inner textile surface and the skin, and the conduction thermal resistance is quite small due to tightness. During the heat loss from the outer surface to the ambient environment, however, the radiation contributes more significantly than the convection heat transfer in a typical indoor environment. Therefore, it indicates that reducing the outer surface emissivity can efficiently suppress the radiative heat loss from the human body. Based on this conception, Cai et al. [76] demonstrated a cotton-based textile with nanophotonic structures that were composed of nanoporous silver and nanoporous PE (nano-Ag/PE) (Fig. 11a, b). It showed a high reflectance of 98.5% on the inner surface to reflect the human body’s radiation and a strongly inhibited thermal emissivity of 10.1% on the outer surface to reduce the heat loss from the textile. Compared to normal textile, this metalized textile enabled a 7.1 °C reduction of the temperature set-point, potentially saving more than 35% of building heating energy (Fig. 11c). Besides, it showed various comparable wearabilities, including wicking rate, water vapor transmission rate, mechanical strength, and great durability against washing. Wu et al. [120] developed a multi-material aerogel fabric, fabricated by coating an Ag layer on an aerogel composite fabric, to realize passive personal heating without any energy input. The lightweight aerogel composite fabric, woven from aerogel composite fibers featuring a multi-scale porous structure, demonstrated exceptional thermal insulation, self-cleaning capabilities, mechanical resilience, and thermal stability. By applying an Ag layer coating, the fabric exhibited both low thermal conductivity and low IR emissivity at 7–14 μm, demonstrating superior thermal insulating performance (Fig. 11d). Consequently, the proposed fabric with a thickness of 1.29 mm could enhance the human body temperature of 5.7 °C in a cold environment, without requiring any energy input (Fig. 11e, f). In addition to silver, other metallic fibers could also exhibit a low IR emissivity for thermal insulation. Larciprete et al. [121] found that textiles composed of steel yarns that determine the low absorption/emission within the MIR range appeared to be suitable for thermal shielding applications. And the thermal properties of the metal composite fabrics could be modified by the content of metal components, metal mesh openness, and layer arrangement with the fabric [122]. Although these metallic fibers could be woven or knitted into textiles, they are always stiff, heavy, and fragile, usually leading to discomfort in wearability. Therefore, it still remains a challenge to address the contradiction between optimal heat transfer performance and excellent wearability through more advanced photonic structure design.

### UV–VIS–NIR and FIR Radiative Heating Textile Materials

#### UV–VIS–NIR Radiative Heating

**Fig. 12:**
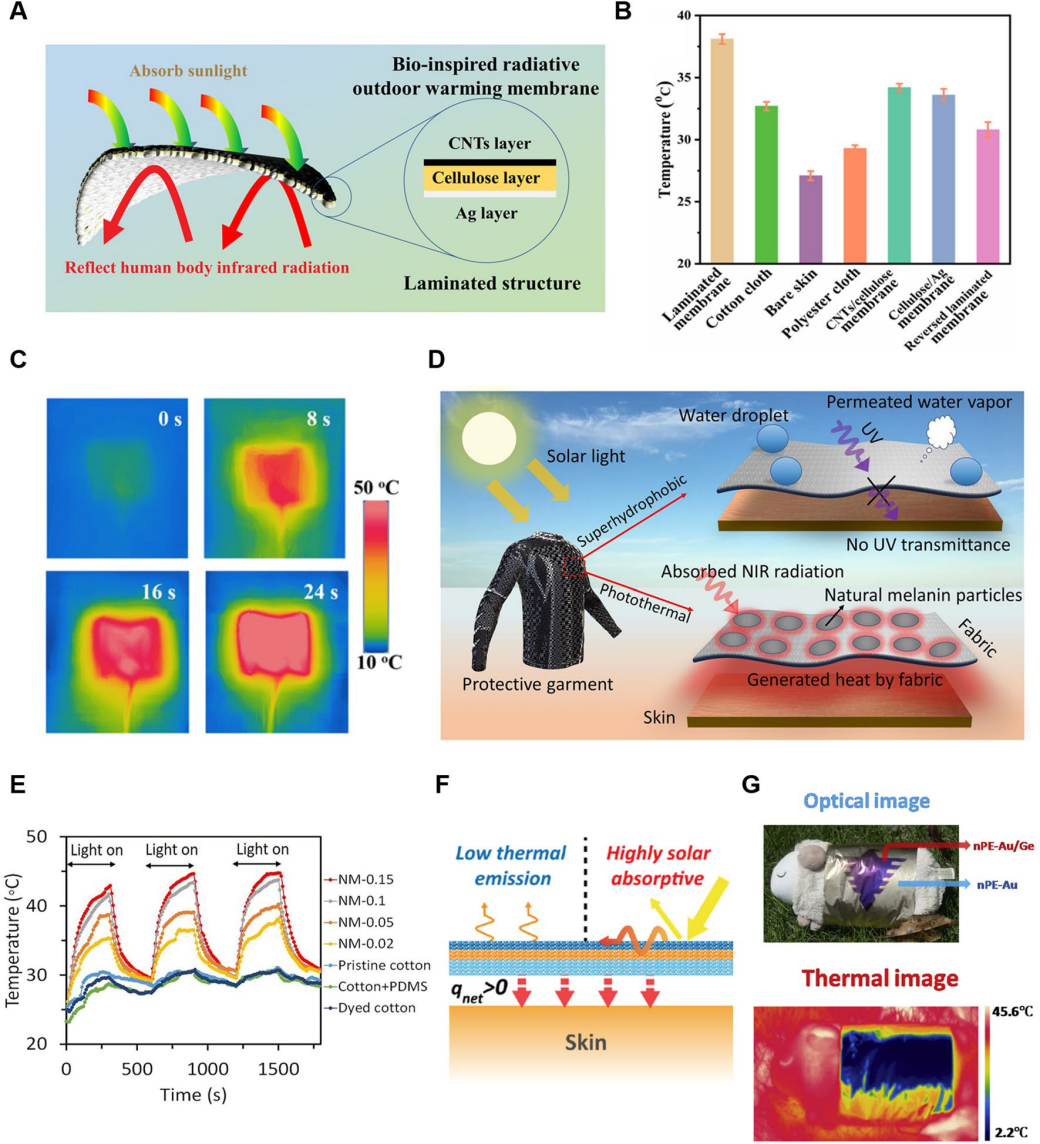
UV–VIS-NIR radiative heating materials. **a** Laminated structure of the biomimetic membrane. **b** Comparison of the temperature for the laminated membrane, cotton cloth, bare skin, polyester cloth, CNT/cellulose membrane, cellulose/Ag membrane, and reversed laminated membrane (condition: the thickness of all samples is about 0.5 mm). **c** Thermal images of the biomimetic membrane supplied with a voltage of 3.7 V [127]. Reproduced with permission. Copyright 2020, American Chemical Society. **d** Schematic illustration of novel functionalities of cotton fabric after coating with NM/PDMS. **e** Surface temperature change of cotton samples over 3 cycles of NIR irradiations [128]. Reproduced with permission. Copyright 2022, Elsevier. **f** Schematics depicting the heating mechanism of the colored textile with simultaneous solar and passive heating abilities. *q*_net_ denotes the net heat flux from the textile into the skin. **g** Optical and thermal images of a toy sheep partly wearing a colored textile with simultaneous solar and passive heating abilities [129]. Reproduced with permission. Copyright 2019, Elsevier

After the absorption by the ozone layer, the solar radiation that reaches the earth’s surface is composed of 5% ultraviolet light, 46% visible light, and 49% near-infrared light. Application of photothermal conversion materials or devices could partially or completely convert the aforementioned light into the required thermal energy [123, 124]. Inspired by the natural animals in extremely cold environments, researchers have designed advanced fiber materials for heating or warming the human body by photothermal conversion. The polar bear’s hairs with a long transparent hollow structure have a smooth external surface and the air can be captured inside the hollow structures as well as among the hairs [125]. The sunlight can be captured by the air inside the hollow structure, and then be transmitted to the black skin for storing the heat by the thick fat layer. In the meantime, the hairs on the pelt could efficiently reflect the thermal radiation from the polar bear’s body [126]. Inspired by the polar bear’s hair structure for adapting to the cold environment, Yue et al. [127] reported a porous Ag/cellulose/CNT laminated nanofiber membrane (Fig. 12a). CNTs with high solar radiation absorptivity were coated on one side of the cellulose basement membrane, and the Ag layer with high IR reflectance was deposited on the other side by magnetron sputtering. Therefore, the biomimetic membrane achieved radiation warming by absorbing the heat input from the sun and blocking the human radiation heat output. Also, the Ag layer could act as a heater to induce a fast thermal response to provide extra warmth under a low supplied voltage. The simulated skin temperature was 5.1 °C higher than that of cotton covered (Fig. 12b). After being electrified under a voltage of 3.7 V for only 24 s, the biomimetic membrane could reach a high temperature of 48 °C from the room temperature of 15 °C (Fig. 12c). Except for the polar bear’s hairs, the cotton surface could be also modified by applying coatings that contain natural melanin (NM) particles extracted from yak hair and PDMS [128] (Fig. 12d). The NM-coated fabrics provided a rapid heating effect under NIR and the temperature increased to 38.4–45.3 °C, which was up to 14.7 °C higher than that of pristine cotton (Fig. 12e). Besides, the NM-coated fabric showed durable superhydrophobicity and UV protection without damaging the natural breathability of cotton fabrics.

In addition, Luo et al. [129] designed an ultra-thin colored textile with a nanophotonic structure made of a PDA-coated nanoporous textile coated with a reflective metal layer of Au and an ultra-thin lossy dielectric layer of Ge for simultaneous solar energy absorption of about 50% and passive radiative heating with a low IR emissivity of about 10% (Fig. 12f). Strong interference induced by the optical coatings enabled broadband selective solar absorption for heating, while the textile maintained low MIR emissivity (< 10%) for passive heating. The artificial skin covered with the textile attained temperatures of 3.8/6.4 °C higher as compared to that covered with a 2-mm-thick black sweatshirt in indoor/outdoor environments (Fig. 12g). Besides, it showed excellent aesthetics, wearability, and manufacturability. In order to develop the photothermal technology on the fabric substrate, Zirconium carbide (ZrC) films were deposited onto polyester fabric by magnetron sputtering [130]. The results showed that the highest far-infrared emissivity of polyester fabric deposited with ZrC was 93.79%, indicating that the far-infrared wavelength could be absorbed by the human body for generating warmth. Meanwhile, the ZrC deposited samples showed a small increase in thermal conductivity with a difference of 0.0611 W m^−1^ K^−1^ and presented a higher photothermal conversion efficiency with a temperature increase of 27.5 °C in 100 s when the thickness of the ZrC film is 1920 nm.

#### FIR Radiative Heating

Far-infrared rays with a wavelength of 6–15 μm can penetrate 2–3 mm into various biological materials and exert strong vibrational and rotational effects at the molecular level, resulting in the enhancement of blood microcirculation and metabolism [131, 132]. The FIR rays can be absorbed by the epidermal layers of the human body, leading to a pleasant thermal warmth sensation. Far-infrared emitting materials are capable of transforming the energy absorbed from sunlight radiation or human body radiation into far-infrared rays with a specific wavelength range, and then remit to the human body [133]. Due to the enhanced blood circulation and promoted recovery of fatigue muscles induced by FIR, much attention has been paid to the development of FIR emissive materials. At present, the most widely used approach for the fabrication of FIR materials is to incorporate germanium and ceramics into the fabric matrix that is in close contact with the human skin. These FIR emissive materials mainly include MgO, SiO_2_, ZnO, CNTs, ZrC, ZrO_2_, and, germanium compounds. Hu et al. [134] fabricated a cotton fabric by depositing the composites of graphene and waterborne anionic aliphatic polyurethane composites through the facile pad-dry-cure process. The coating process improved the FIR emissivity up to 91.10% in the wavelength range of 4–18 μm. In addition, the coated fabric showed excellent performance in ultraviolet blocking, electrical conductivity, and stability against laundering. Recently, attention has been paid to the preparation of the infrared radiation heating fabric by coupling electrical heaters and infrared particles. Qiu et al. [135] presented a highly flexible, efficient, and sandwich-structured infrared radiation heating fabric (IRHF). Two layers of PET fabric were chosen as the top and bottom substrates, while one sandwiched layer of carbon nanofibers connected with conductive copper electrode sheets served as the main working layer in the composite. And several inorganic nanoparticles, including SiO_2_, TiO_2_, and tourmaline, were incorporated into the high conductive carbon nanofibers developed by the electrospinning process and a carbonization approach. The permanent spontaneous polarization of both carbon nanofibers and infrared radiation nanoparticles could facilitate an improved current in the heater by creating an additional electrical field, leading to a fast electrothermal response and favorable heat preservation. The constructed IRHF achieved an increase in the temperature to 43 °C from room temperature in 1 min under a voltage of 30 V, with electrothermal conversion efficiency up to 78.99%. In addition, the FIR radiative heating performance was related to the fiber shape. Li et al. [136] numerically and experimentally explored the interaction between fiber shapes and FIR performance. It showed that compared with the reference circular fiber, a non-additive triangular PA fiber exhibited higher performance of FIR absorption and emission because the triangular PA fiber afforded a higher probability to facilitate large optical path differences. Textiles woven with the triangular PA fiber achieved a remarkable emissivity of 91.85% and a temperature difference of 2.11 °C, which was obviously superior to the reference circular fiber.

## Dynamic Textiles for Dual-Mode of Radiative Cooling and Heating

Dynamic thermal regulation is attracting heightened interest across a variety of thermal climates and working conditions. Traditional materials and structures, with their singular cooling or heating functions, are proving inadequate in meeting the thermal comfort requirements of increasingly complex and dynamic environments. In this section, we will explore the most recent advancements in dynamic textiles specifically designed for dual-mode thermal regulation. In the sections that follow, we will present three distinct categories of materials: dual-mode textiles composed of a bilayer with differing emissivities, biomimetic materials inspired by animals possessing adaptive thermal regulation functionality, and dynamic responsive materials that react to external stimuli.

### Dual-Mode Textile Composed of a Bilayer Emitter

**Fig. 13:**
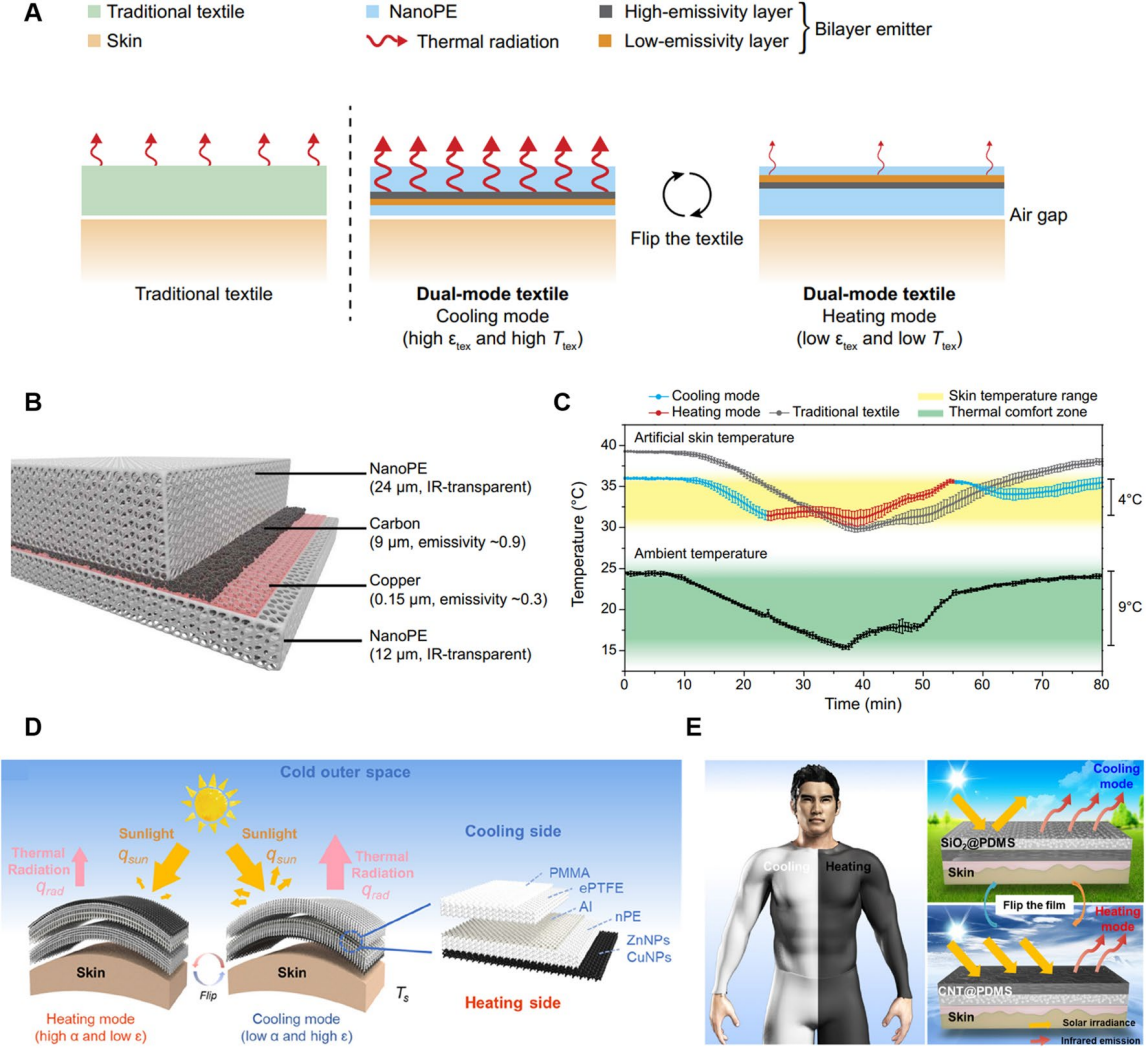
Schematic of working principle, composition structure, and thermal regulation performance of dual-mode textile. **a** Comparison of traditional textiles and dual-mode textiles. **b** Layered structure of the dual-mode textile. **c** Real-time thermal measurements of dual-mode and traditional textiles under varying ambient temperatures [137]. **d** Working principle and the structural component of the Janus textile designed by Luo et al. [139]. Reproduced with permission. Copyright 2021, American Chemical Society. **e** Schematic illustration of the working principle of the Janus film fabricated by Dai et al. [140]. Reproduced with permission. Copyright 2022, American Chemical Society

According to the radiative heat transfer equation, the emissivity and temperature of the emitter surface are crucial factors affecting heat transfer performance. Therefore, to achieve both passive heating and cooling, a bilayer emitter with different emissivities on each side is necessary for the single textile. It is reported that the emissivity of the outer textile surface would play a more significant role than that of the inner surface because the radiative heat transfer between the outer surface and the ambience is more dominant than that between the inner surface and the human body skin [76]. Inspired by this conception, Hsu et al. [137] designed a dual-mode textile composed of a bilayer emitter (Carbon and Cu) embedded inside an IR transparent nanoPE layer with an asymmetric thickness on each side (Fig. 13b). The bilayer emitter controlled the emissivity, while the nanoPE thickness controlled the temperature of the emitter surface. In cooling mode, the high emissivity layer turned toward the environment, and the thickness of nanoPE between the emitter and the human skin was small for ensuring efficient thermal conduction between them and increasing the emitter temperature (Fig. 13a). When the textile was flipped in heating mode, the low emissivity side faced out and the emitter-to-skin distance increased, leading to lower thermal conductance and reduction of heat loss. This could expand the thermal comfort zone by 6.5 °C. Further, numerical fitting of the data predicted 14.7 °C of comfort zone expansion for dual-mode textiles with large emissivity contrast (Fig. 13c). Therefore, it is feasible to achieve passive cooling and heating for the same textile that consists of different IR emissivity components and can control the emitter-to-skin distance. In addition, a multifunctional Janus membrane with a sandwich structure by vacuum filtrating ultralong MnO_2_ nanowires and Cu nanowires sequentially on the basement membrane of cellulose fiber@Layered Double Hydroxide (LDH) [138]. The obtained Janus membrane presented an asymmetrical emissivity of IR radiation for on-demand personal thermal management: the low emissivity layer (Cu nanowires layer) faced outside to hinder the human radiation, and the high emissivity layer (cellulose@LDH layer) turned outside to enhance the human radiation. Besides, it showed excellent breathability, flexibility, interfacial compatibility, and antibacterial activity for wearability. For outdoor personal thermal management with uncontrollable sunlight and intense temperature fluctuations, Luo et al. [139] reported an eco-friendly passive nanostructured Janus textile that could harvest energy from the sun and the outer space for optional localized heating and cooling (Fig. 13d). The heating side manifested low IR emissivity of about 16% and high solar absorptivity over 80%, while the cooling side showed high IR emissivity of about 87% and solar reflectance over 90%. Compared to conventional heating/cooling textiles, this Janus textile enabled a skin simulator temperature increase/decrease of 8.1/6 °C, respectively, under sunlight exposure. Meanwhile, it allowed continuous electricity generation with thermoelectric modules. Also, Dai et al. [140] fabricated a Janus film that integrated two opposite requirements of heating and cooling by a combination of SiO_2_ and CNT embedded into PDMS (Fig. 13e). Overall, the Janus materials with asymmetric IR radiation properties could be a potential candidate for controlling IR emissivity to help humans adapt to the changing environment [141].

### Biomimetic Material Inspired by Natural Animals

**Fig. 14:**
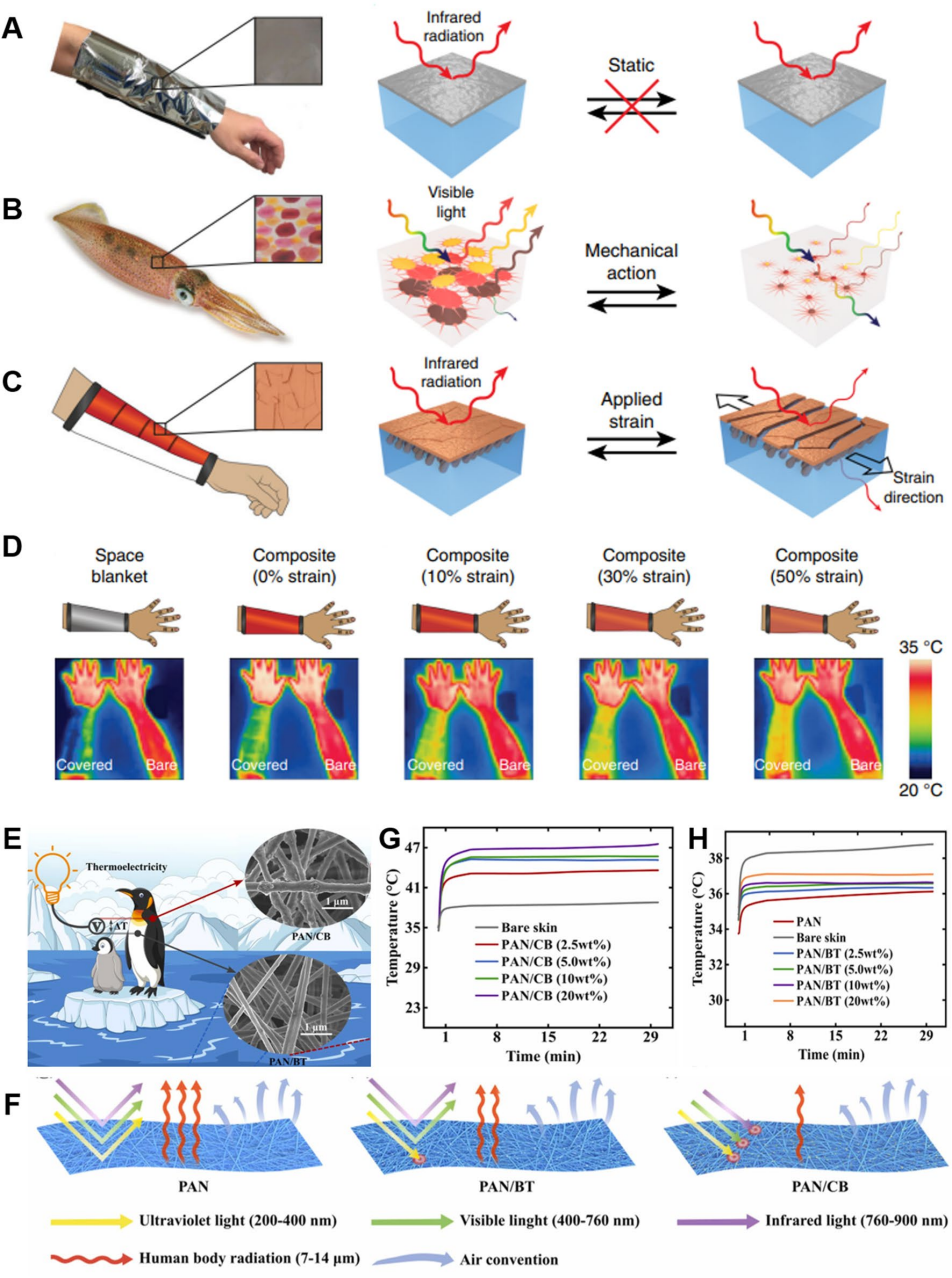
Bioinspired design of thermoregulatory composite materials. **a–c** Schematic of radiation regulation for a space blanket on a human arm, the squid skin, and the composite material on a human arm in a wearable (sleeve) configuration. **d** Infrared camera images of a forearm covered with a space blanket-based sleeve and a composite-based sleeve under strain varying from 0 to 50% [150]. **e** Penguins-inspired dual-temperature-regulation materials. **f** Schemes of light absorption, reflection, IR transparency, and air convection of PAN, PAN/BT, and PAN/BC nanofibers. **g**–**h** Temperature variation of simulative skin when PAN/CB and PAN/BT nanofibers were used as heating or cooling materials with content variation of CB/NPs and BT/NPs under the radiation of a solar simulator for 30 min [151]. Reproduced with permission. Copyright 2019, Elsevier

Many natural species have evolved elegant strategies to manipulate IR radiation for heating and cooling purposes. Inspired by the fascinating dynamic color-changing skin of many natural animals like chameleons and cephalopods, some investigations have been conducted on the design and fabrication of dynamic materials or structures that can change the surface color and emissivity under different circumstances. The dynamic color-changing skin of coleoid cephalopods (squid, octopuses, and cuttlefish) represents a judicious source of inspiration for next-generation adaptive thermal management systems [142, 143]. For example, the squid skin is composed of multiple layers, one of which contains embedded red, yellow, and brown chromatophore organs (Fig. 14b). This highly natural structure facilitates the pigment cells to be dynamically switched by the muscle cells between contracted point-like and expanded plate-like states, thus regulating the local coloration and changing the transmission of light through the skin [144, 145]. Therefore, the unique structure and fascinating function of cephalopod skin have inspired the advent of various unconventional color-changing technologies, including biomimetic soft active surfaces, optoelectronic displays, stretchable electroluminescent materials, electromechanochemically responsive elastomers, and adaptive infrared camouflage [146–149].

As one example, Leung et al. [150] developed a composite material with tunable thermoregulatory properties by leveraging the static infrared-reflecting design of the space blanket and drawing inspiration from the dynamic color-changing ability of squid skin. The composite material contained two layers with the bottom of a soft and stretchable IR transparent polymer matrix that emulated the chromatophore-containing transparent dermal layer of squid, and the top layer of an IR reflecting metal structure that emulated the embedded chromatophore organs. Without mechanical actuation, the composite could nearly reflect all the incident IR radiation (Fig. 14c). With mechanical actuation, the metal structure spread apart and the bottom polymer matrix would be exposed outside, resulting in the transmission of IR radiation. Based on the fabricated material, the custom-designed sleeves were prepared for the demonstration of a wearable PTM system. The composite-based sleeves blocked the IR radiation like a space blank without any mechanical strain, while more radiation was transmitted through the sleeves under an applied strain when the temperature increased (Fig. 14d). The designed material demonstrated an on/off switching ratio of about 25 for the transmittance, regulated a heat flux of about 36 W m^−2^ with an estimated mechanical power input of about 3 W m^−2^, and featured a dynamic environmental set-point temperature window of about 8 °C.

Penguins serve as a prime example of efficient body temperature regulation in extremely cold environments by adjusting their body orientation toward sun irradiation. When they feel cold, they tend to face their black backs toward the sun to absorb thermal energy. Conversely, their white abdomens can reflect a portion of sunlight radiation when they do not require excessive heat. Drawing inspiration from the dual-temperature regulation function of penguins, Ye et al. [151] developed a biomimetic dual-functional composite, which composite includes polyacrylonitrile/barium titanate nanoparticles (PAN/BT NPs) as cooling materials and polyacrylonitrile/carbon black nanoparticles (PAN/CB NPs) as heating materials, prepared using the electrostatic spinning method. In this composite, BT NPs and CB NPs are uniformly dispersed in PAN nanofibers (Fig. 14e), and the PAN/BT cool-type nanofibers exhibited a high solar reflectivity of 89.59% and a MIR transmission exceeding 95.16%, while the PAN/CB heat-type nanofibers showed a high solar absorptivity of 93.5% and a poor middle-infrared transmission below 50% (Fig. 14f). Under 1 Sun sunlight illumination for 30 min, the temperature of a skin simulator could decrease 2.5 °C for the cooling side and increase by 8.7 °C for the heating side (Fig. 14g, h).

### Dynamic Responsive Change of Fiber/Yarn Structure

Improving the structure of responsive thermal regulation systems for personal thermal management is crucial for achieving the necessary thermoregulation. The temperature or humidity variation could stimulate the inner morphology change of the textiles, such as the pore size, the thickness, and the fiber structure. These responsive textiles could achieve an adaptive thermal regulation function for PTM under diverse conditions. Most of the thermally responsive fibers are kind of shape memory polymers, whose macroscopic properties (*i.e.*, color, shape, and state) are capable of varying with any change in light, heat, and electricity. The shape memory polymers have been manufactured in previous studies, showing that the fibers could be stretched up to 200%, and the internal structure of loose knots was able to transform into tight knots in 20 s [152]. In addition, temperature memory polymers and liquid crystal elastomers are also used for thermoresponsive materials [153, 154]. Although advanced thermoresponsive textiles can be developed by coating the above functional materials on traditional fabrics, there are remaining addressed problems. For example, certain materials could only achieve one-way shape memory at a constant temperature, or they are not sufficient to sense the little temperature variation of the human body due to the weak sensitivity.

**Fig. 15:**
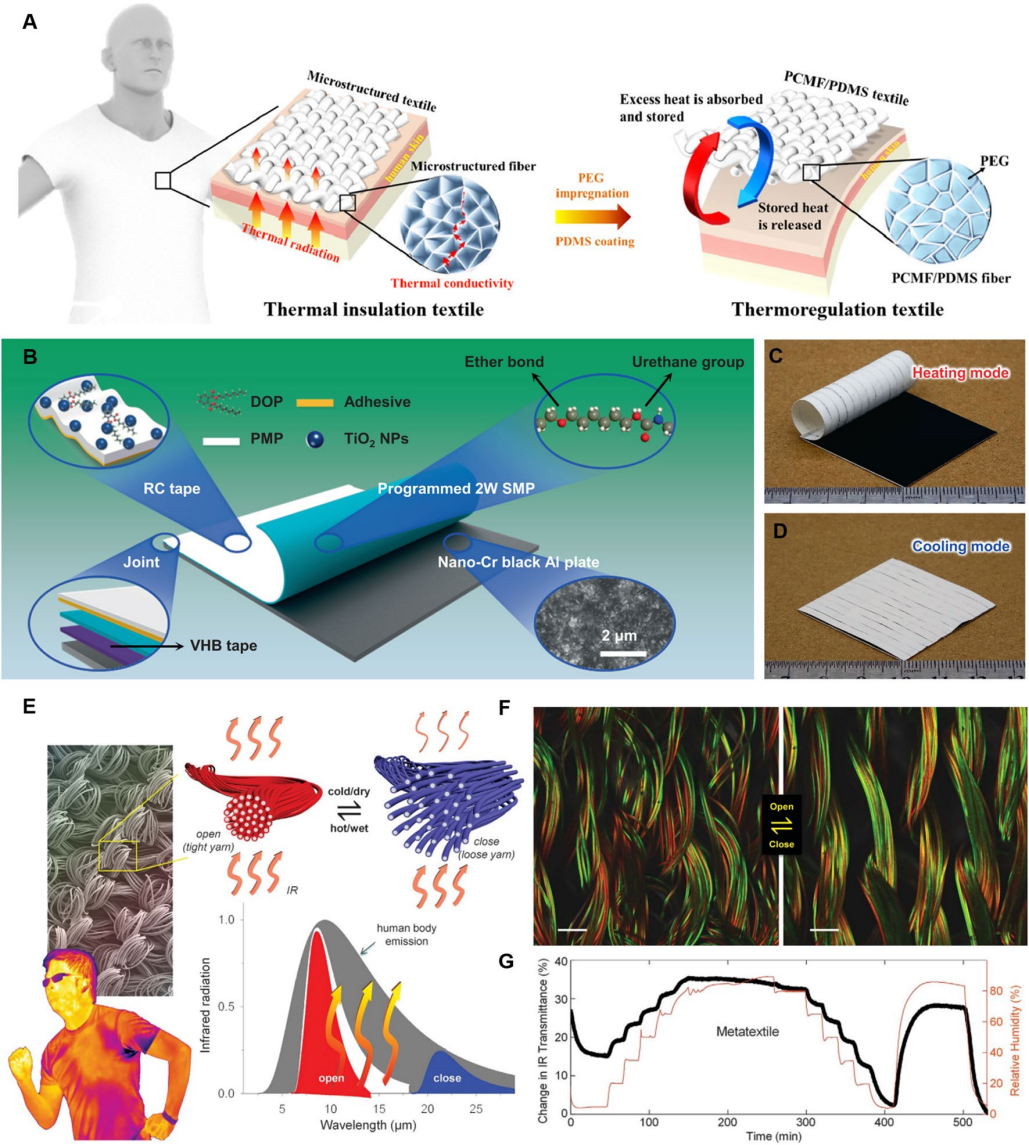
Dynamic responsive materials for dual-mode thermal regulation. **a** Illustration of the working principle and fabrication process of thermal insulation and thermoregulation textiles [20]. Reproduced with permission. Copyright 2020, American Chemical Society. **b** Structural illustration of a thermally actuating dual-mode thermal management device. The nano-Cr black Al plate serves as the solar collector for solar heating. The functional layer for radiative cooling in the RC tape is composed of a DOP-modified PMP matrix and TiO_2_ NPs fillers. A narrow strip of VHB tape, used as the sole joint part between solar heating and radiative cooling layers, preserves the maximum effective area for dual-mode thermal management. The inset of SEM image shows that nano-chromium oxide powders are uniformly distributed on the aluminum plate. **c**, **d** Optical images of the dual-mode device in heating and cooling modes [155]. **e** Design principles of the IR gating textiles. Each yarn knitted into the textile is composed of multiple metafibers that contain IR-active nanostructures. **f** Confocal fluorescent microscopy images showing the knitted fabric in the closed state (left) (low humidity) and the open state (E) (high humidity). To illustrate the side-by-side bimorph structure of the fibers, the hydrophilic cellulose component was dyed with an aqueous solution of rhodamine B (red), and the hydrophobic triacetate component was dyed using coumarin 6 (green) from a mixed organic solvent. **g** The IR transmittance change over the atmospheric transmission window (8–14 μm) of IR gating metatextile (black line) with different relative humidity profiles [72]. Reproduced with permission. Copyright 2019, American Association for the Advancement of Science

Wu et al. [20] demonstrated a skin-friendly personal insulation textile and a thermoregulation textile that can perform both passive heating and cooling using the same piece of textile (Fig. 15a). The insulation textile material was made up of biomaterial microstructured fibers, which offered excellent thermal insulation, low thermal emissivity, and good dyeability, thereby reducing human radiation loss. By filling with biocompatible polyethylene glycol (PEG) and coating them with PDMS, the phase change microstructured fibers (PCMFs) could become a thermoregulation textile that exhibited good water hydrophobicity, high mechanical robustness, and high working stability. It demonstrated slower heating and cooling rates than the polyester glove in 50 and 10 °C isothermal environments and could significantly extend the skin comfort time up to 70 and 115 s, respectively. Zhang et al. [155] reported an intelligent, zero-energy dual-mode radiative thermal management device, which was capable to automatically switch modes depending on the ambient temperature. As shown in Fig. 15b, this dual-mode device consisted of three functional layers, a radiative cooling layer, a temperature-sensitive actuating layer, and a solar heating layer. The essence of zero-energy dual-mode strategy was based on the transformation of required high-selectivity spectral characteristics in the temperature control system. In heating mode, the radiative cooling layer automatically coiled to maximum exposure of the solar heating layer, which had high solar absorptivity and low infrared emissivity, resulting in the absorption of solar radiation and the suppression of infrared radiation loss (Fig. 15c). When the cooling mode was needed, the radiative cooling layer completely covered the solar heating layer, minimizing solar absorption and preventing an increase in internal energy. Concurrently, the high MIR emission in the wavelength range of 8–13 μm could directly transfer heat into outer space, reducing undesired infrared radiation from air and surrounding environment (Fig. 15d). Consequently, the device could achieve about 859.8 W m^−2^ of average heating power (about 91% of solar thermal conversion efficiency) in cold conditions and about 126.0 W m^−2^ of average cooling power in hot conditions, without consuming any external energy throughout the process. Zhang et al. [72] produced a metatextile with smart and dynamically adaptive IR optical properties by incorporating tunable electromagnetic interactions at the fiber level to directly regulate thermal radiation from the human body. Each yarn was composed of a bundle of metafibers, which were obtained by adding the metaelement (CNTs) to the bimorph polymer textile fibers composed of hydrophobic triacetate and hydrophilic cellulose (Fig. 15e). This IR-adaptive textile could respond directly to temperature/ relative humidity changes of the human skin. Under humid or hot conditions, the yarn would collapse, making the metaelements on neighboring fibers closer together to induce resonant electromagnetic coupling (Fig. 15f). And the coupling behavior could shift the emissivity of textile to better match the thermal radiation emitted from the human body, thus enhancing heat transfer efficiency. When cold or dry, the yarn would respond oppositely to reduce heat dissipation. In other words, it is possible to effectively gate (i.e., “open” and “close”) the IR radiation through the textile in response to ambient change (Fig. 15g). Meanwhile, the change of interpore sizes could regulate the conventional heat transfer, such as convection, conduction, and evaporation, synergistically enhancing IR gating effects.

## Conclusions and Outlook

**Fig. 16:**
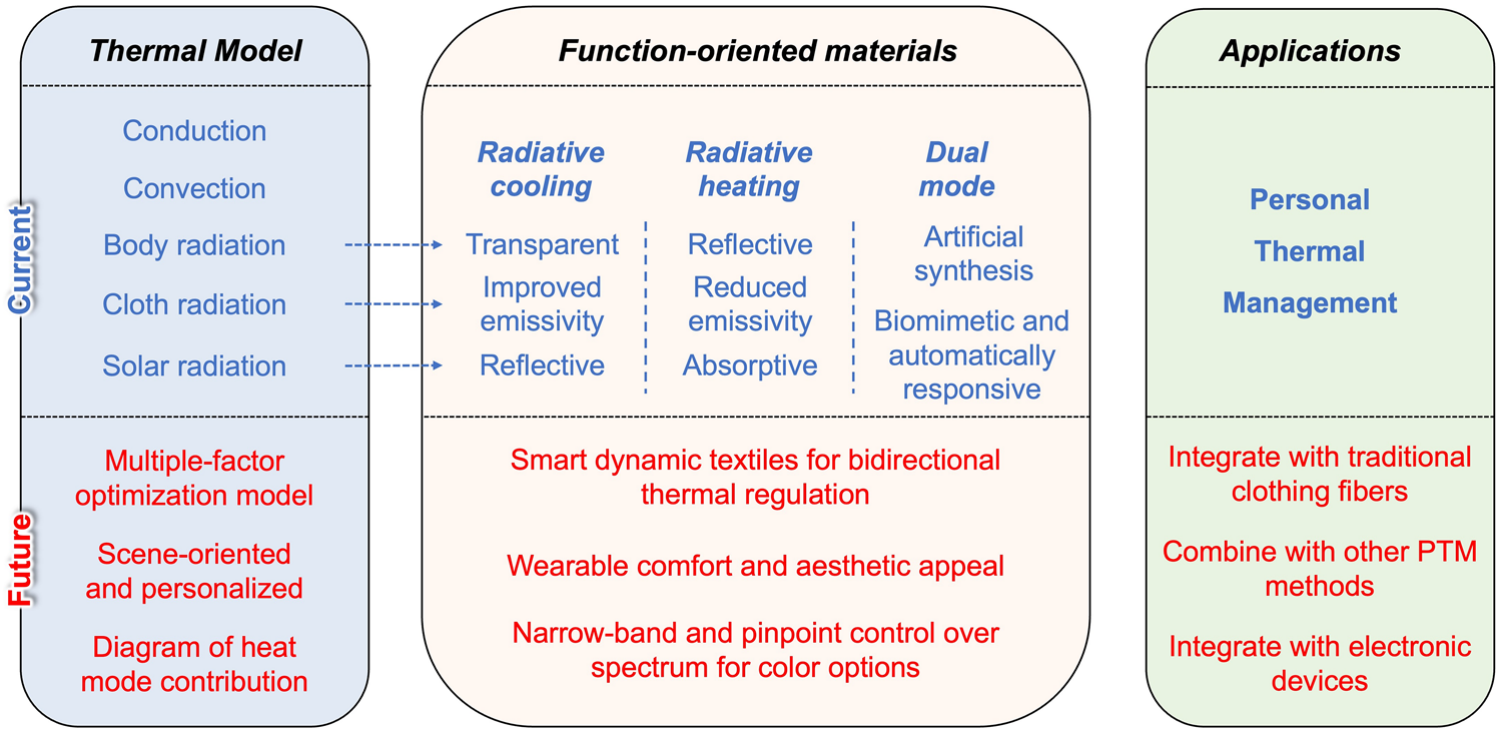
Schematic summary of conclusions and outlook for PRTM

In this paper, we provide a comprehensive review of advanced textile materials for PRTM, exploring the interaction between thermal models, function-oriented design principles, and practical applications including radiative cooling, radiative heating, and dual-mode cooling and heating. As outlined in Fig. 16, we start by introducing the radiative heat transfer models, followed by a summary of material design principles based on optical properties for various thermal regulation requirements. For radiative cooling, we discuss materials in the following sequence: IR transparent materials based on nanoPE, solar reflective materials based on metal compounds, and high IR emissive materials at ATSW. Similarly, for radiative heating, we summarize IR reflective, low IR emissive, and solar absorptive materials. Lastly, we delve into dynamic textiles for dual-mode cooling and heating, which encompass artificial synthesis and bionic fabrication inspired by nature. These significant advancements in PRTM have contributed to the evolution of PTM concepts and strategies. This overview of progress in PRTM will enhance our understanding of the current research and design status, regulation mechanisms, and perspective exploration in this field. Following a comprehensive summary and analysis of the research, we will spotlight future trends in advanced textile materials for PRTM. These trends will be discussed from the perspectives of thermal models, functional requirements, and practical applications:

1. The actual performance of thermal regulation is determined by a combination of factors, including the clothing material, environmental conditions, and individual characteristics. However, there is currently a lack of a comprehensive optimization model for designing specific materials based on personal heat transfer characteristics, such as metabolic heat generation rate, temperature requirements for different age groups, and sweat amount, as well as environmental conditions like solar irradiance intensity, ambient temperature, and relative humidity. Developing such an optimization and scene-oriented model is crucial for achieving personalized, on-demand thermal comfort regulation.
2. Previous studies have shown that radiative heat transfer in PTM is highly effective in regulating thermal comfort without the need for extra energy consumption. However, the efficiency of radiative heat transfer depends on the temperature difference between the cloth surface and the surrounding atmosphere, making it weather dependent. Moreover, the contribution of radiation to personal thermal comfort regulation under varying atmospheric conditions remains poorly understood, especially in scenarios where there is a minimal temperature difference and the body is perspiring in high temperatures during summer. Consequently, a guiding diagram illustrating the contributions of various heat transfer modes (such as conduction, convection, radiation, or sweat evaporation) under different atmospheric conditions (like solar radiation intensity, air temperature, and wind speed) would be advantageous. This would aid in selecting the most suitable heat transfer mode for practical applications in thermal comfort regulation.
3. The rapid advancement of bionic engineered materials, inspired by nature, has led to a desire for smart dynamic textiles that possess reversible IR-adaptive properties. These textiles are sought-after for achieving bidirectional thermal regulation, particularly in response to fluctuating weather conditions.
4. In addition to thermoregulation efficiency, several other factors are crucial in determining personal wearable comfort and aesthetic appeal for commercial applications, including air permeability, sweat management, flexibility, durability, cloth color, and safety considerations when additional materials are incorporated. Importantly, achieving narrow-band and pinpoint control over the visible spectrum is essential not only for promoting thermoregulation, but also for providing a wide range of textile color options.
5. Although various advanced functional materials have been designed and applied to enhance the solar or human radiation spectrum behaviors in PTM, there still exists a gap when it comes to integrating these materials with traditional clothing fibers in a way that considers both thermoregulation efficiency and long-term wearability in practical applications. The advantages and limitations of commonly used clothing fibers are summarized in Table 6. Natural fibers are known for their excellent moisture absorption and air permeability but tend to lack shape retention, resulting in issues like wrinkling, shrinking, and deforming. On the other hand, synthetic fibers offer high strength and durability but often fall short in terms of moisture management and air permeability. Therefore, there is a need to further explore the manufacturing and coupling technologies that can effectively integrate the radiative heat transfer performance of functional materials with commonly used clothing fibers.
6. The effective enhancement of thermal comfort regulation can be achieved through the combination of radiative heat transfer with other PTM methods. Advanced textiles can be integrated with other flexible electronic devices, such as wearable batteries and moisture/temperature sensors, to create next-generation smart textiles. These smart textiles offer numerous functions, including self-powering, automatic sensing, computing, and regulation for personal thermal management.
7. Currently, the temperature distribution, typically obtained by thermocouples or an infrared thermal camera, on simulated skin or a real person’s body is commonly used to quantitatively evaluate and compare the performance of personal radiative thermal management systems. Additionally, the optical parameters (transmittance, reflectance, and emissivity) of textiles materials within the desired spectrum are compared to evaluate these systems, as outlined in Tables 3, 4 and 5. However, there are currently no standardized specifications, such as dimensionless parameters or indexes, for evaluating radiative thermal performance under universally accepted, controlled conditions. Therefore, the development of standardized specifications for personal radiative thermal measurements and evaluations is of paramount importance for future research.

**Table 3:** Summary of materials, fabrications, thermal regulation results, advantages, and limitations of different radiative cooling materials

References	Structures/materials	Fabrication methods	Optical properties and thermal regulation results	Advantages	Limitations
*IR transparent textile materials*					
Hsu et al. [71]	NanoPE	Microneedle punching, point wielding, and lamination	Average IR transmittance of 96% and 77.80% for PDA-nanoPE-mesh. Lower the simulated skin temperature up to 2.7 ℃ for nanoPE and 2.0 ℃ for PDA-nanoPE-mesh	Sufficient air permeability, water-wicking rate, and mechanical strength for wearability	Uncomfortable to wear due to its plastic-like nature
Peng et al. [80]	Uniform and continuous nanoPE microfiber	NanoPE microfiber was extruded with paraffin oil and knitted/woven into fabric	Average IR transmittance over 70%. Lower the simulated human skin temperature by 2.3 ℃	Excellent water-wicking rate, wearability, and durability	N/A
Cai et al. [81]	PE	Nanoparticle-mixed PE composites were extruded and knitted into textiles	Average IR transmittance of 80%. Lower the simulated skin temperature by 1.6–1.8 ℃	Colored textile, good water-wicking ability, mechanical strength, and excellent stability against washing	N/A
*Solar reflective textile materials*					
Wong et al. [98]	TiO_2_-coated cotton	Calcination treatments	Solar reflectance of 84.80%. Lower the surface temperature by 3.9 ℃	Excellent air permeability and washability	Complicated fabrication
Panwar et al. [99]	TiO_2_-SiO_2_ Janus-coated cotton	Pickering emulsion method and exhaustion method	NIR reflectance of 79%. Lower the surface temperature by about 3 ℃ than uncoated cotton	Easily applied to a flexible textile substrate	Complicated fabrication
*Solar reflective and IR emissive textile materials*					
Wei et al. [69]	Al_2_O_3_-CA-coated textile	Dip-coated onto textiles or Mayer rod-coated on PET substrate	Solar reflectance of 80.1% and IR emissivity of 97%. Lower the simulated skin temperature of 2.3–8 ℃ and avoided the overheating of actual human skin by 0.6–1.0 ℃	Scalable, low cost	Slow fabrication process, poor wearability, and dyeability
Zhang et al. [100]	PDMS encapsulating Al_2_O_3_	Microstamping method	Solar reflectance of about 95% and IR emissivity over 96%. Lower the temperature up to 5 ℃	Great flexibility and superior strength	Poor air permeability and wearable discomfort due to its film nature
Wang et al. [74]	PVDF/TEOS with SiO_2_ on the surface	Electrospinning and emulsion deposition	Solar reflectance of about 97% and average IR emissivity over 96%. Lower the temperature up to 6 ℃	Scalable, low cost, great flexibility, and superior strength	Poor air permeability and wearable discomfort due to its film nature
*Solar reflective and IR transparent textile materials*					
Cai et al. [51]	ZnO NPs embedded into nanoPE	Melt-pressed into a thin film	IR transparency of 80% and solar reflectance over 90%. Enable the simulated skin to avoid overheating by 5–13 ℃	Scalable	Surface hydrophobicity and poor moisture-wicking
*Improving IR emissive textile materials*					
Zeng et al. [18]	TiO_2_-PLA metafiber with PTFE laminated on the top layer	Metafiber was extruded, melt spinning and drafting, and weaved into textile, finally laminated with PTFE	Average IR emissivity of 94.5% and solar reflectance of 92.4%. Lower the surface temperature over 3 ℃ than cotton	Superior tensile properties and mechanical strength, waterproofness with air permeability, durability, and washing resistance	N/A
Song et al. [107]	PA/PVDF/PE	Electrospinning	IR emissivity of about 90% and solar reflectance of 90.22%. Lower the body temperature by 6.5 ℃	Scalable, lightweight, high wearable comfort, and economical	N/A
Song et al. [108]	Porous PE and PEO	Melt mixing, melt spinning, cold drawing, and weaving	IR emissivity of 90.97% and solar reflectance of 93.77%. Avoided the human body overheating by 6.8 ℃	Scalable, superlight, flexible, moisture-permeable, waterproof, excellent tensile strength, and UV protection	N/A
Gu et al. [109]	CF@Zn–Al LDHs/CF@ZNR	Hydrothermal method, vacuum filtration, and magnetron sputtering	Maximum IR emissivity of 98% and IR transmittance of 83.0%	Antibacterial, ultraviolet resistant, good flexibility, and breathability	Complicated fabrication
Jeong et al. [111]	PDMS, SiO_2_, and Ag	Photo-lithography, sputtering, spin coating	Average emissivity of 98% and solar reflectance of 95%. Cooling by 6.2 ℃ below the temperature of ambient air	Gradient refractive index effect	Complicated fabrication, and wearable discomfort

**Table 4:** Summary of materials, fabrication, thermoregulation performance, advantages, and limitations of different radiative heating systems

References	Structures/materials	Fabrication methods	Results	Advantages	Limitations
*IR reflective textile materials*					
Hsu et al. [114]	AgNWs embedded cotton cloth	Dip-coating method	Single-layer AgNW network with simulated IR reflectance of 95%. Provide 21% more thermal insulation	Vapor permeable, excellent thermal insulation, durability, and capable of Joule heating	High cost and metal oxidation
Yu et al. [115]	AgNWs and PDA-coated cotton	Dip-coating method	Average MIR to FIR reflectance of 86%. Allowed Joule heating to increase from 22 to 40 °C in 1 min	Durability, washability	Poor substrate adhesion
Yue et al. [116]	Ag NPs/cellulose fibers	Silver mirror reaction	Maximum IR reflectance over 50%. A decreased temperature of 1.9–2.3 °C for IR images due to reflected radiation	Good breathability and excellent antibacterial ability	Wearable discomfort due to its membrane nature
Liu et al. [112]	PDMS-SiO_2_/CA-coated Ag	Coating method	Average transmittance close to 0%. A decreased temperature of 1.3–1.6 °C for IR images due to reflected radiation	Good breathability, waterproofness, antibacterial ability, and capable of Joule heating	Metal oxidation
Hazarika et al. [117]	WKF coated by Cu-Ni NWs and rGO	Hydrothermal method	Average IR reflectance over 98%. Cu_3_Ni_1_-WKF/PDMS provided 43% more thermal insulation than bare WKF/PDMS	Sufficient breathability and high durability	Sensitivity to moisture and oxygen
Hazarika et al. [119]	WKF/Ag@MoxFe_1−*x*_Se/MXene/PDMS	Hydrothermal method and spin coating	MIR reflectance of 97.4% and UV–VIS-NIR absorptivity of 87.10%	High tensile strength, high self-powered heating efficiency, good breathability, and high durability	Complicated fabrication
*Reducing IR emissive textile materials*					
Cai et al. [76]	nanoPE and nanoporous Ag-coated cotton	Electroless plating metallic film onto nanoPE and laminated with cotton	IR reflectance of 98.5%, inhibited thermal emissivity of 10.1%. Enabled a 7.1 °C reduction of the set-point	Lightweight, good breathablity, high durablity, good washability, and good colorability	N/A
Wu et al. [120]	Aerogel composite fabric coated with an Ag layer	Continuous coaxial wet spinning process	IR emissivity of 22.2–45.6%, improve the human body temperature of 5.7 °C (thickness of 1.29 mm)	Lightweight, self-cleaning, good breathability, high mechanical and thermal stability	Metal oxidation
*UV–VIS–NIR radiative heating textile materials*					
Yue et al. [127]	Porous Ag/cellulose/CNT laminated nanofiber membrane	CNT foam finishing process and magnetron sputtering	Average human body IR reflectance of 76% for the Ag layer, solar absorptivity over 90% for the CNT layer. The simulated skin temperature was 5.1 °C higher than that of cotton covered	Possess high porosity, hydrophilicity, breathability, flexibility, and mechanical stability	N/A
Luo et al. [129]	Au and Ge deposited onto PDA-coated nanoPE textile	Vacuum magnetic sputtering	Solar absorption of about 50% and low IR emissivity of about 10%. Higher temperatures of 3.8 °C /6.4 °C than a black sweatshirt	Excellent aesthetics, wearability, manufacturability, and easy manufacturability	Discomfort wearability
Xu et al. [130]	ZrC coated on polyester fabric	Magnetron sputtering	FIR emissivity of 93.79%. An increased temperature by 27.5 °C in 100 s	High photothermal conversion efficiency	Discomfort wearability
*FIR radiative heating textile materials*					
Hu et al. [134]	Graphene and polyurethane deposited onto cotton	Pad-dry-cure process	FIR emissivity up to 91.10%	Excellent ultraviolet blocking, electrical conductivity, and stability against laundering	N/A
Qiu et al. [135]	PET-carbon nanofiber-inorganic NPs	Electrospinning process, carbonization, and lamination	Increasing temperature to 43 °C from room temperature in 1 min with electrothermal conversion efficiency up to 78.99%	High dispersing efficiency, air permeability, and heating stability	Complicated fabrication process and restricted flexibility

**Table 5:** Summary of materials, fabrication, thermoregulation performance, advantages, and limitations of dynamic different radiative materials

References	Structures/materials	Fabrication methods	Results	Advantages	Limitations
Hsu et al. [137]	Bilayer emitter of carbon and Cu embedded in nanoPE	Doctor blade coating, magnetron sputtering, and stacking	Expand the thermal comfort zone by 6.5 °C	Dual-mode, good air permeability, water-wicking rate, and mechanical strength	Complicated fabrication process
Yue et al. [138]	Cu/MnO_2_/cellulose@LDH membrane	Vacuum filtration technology	Average low emissivity of 43.60% for the Cu layer and high emissivity of 97.30% for the cellulose@LDH layer	Good breathability and flexibility, interfacial compatibility, and antibacterial activity	Metal oxidation and discomfort wearability
Luo et al. [139]	PMMA/ePTFE/Al/nanoPE/ZnNPs/CuNPs	High-vacuum magnetic sputtering and spraying coating	Increased/decreased of 8.1/6 °C on heating/cooling mode	Electricity generation	Complicated fabrication
Leung et al. [150]	IR transparent polymer matrix (styrene-ethylene-butylene-styrene) and IR reflecting structure (Cu)	Electron-beam deposition, spin-casting, and delamination	Dynamic environmental set-point temperature window of about 8 °C	Scalable, stretchable, flexible, good reversibility, tuneability, and stability	Poor air permeability
Ye et al. [151]	PAN/BT NPs for cooling and PAN/CB NPs for heating	Electrospinning method	Cooling: solar reflectivity of 89.59% and MIR transmission > 95.16%, decrease 2.5 °C; Heating: solar absorptivity of 93.5% and MIR transmission < 50%, increase 8.7 °C	Flexible, self-powered, washability, and superior electoral output performance	
Wu et al. [20]	Phase-change microstructured fibers (PCMFs) coating with PDMS	Freeze-spinning method	IR emissivity of 0.059–0.231, MIR emissivity of 0.149 at 9.5 μm for one-layer textile, slow heating and cooling rates than polyester	Good water hydrophobicity, high mechanical robustness, and high working stability	Restricted phase change temperature
Zhang et al. [155]	Nano-Cr black Al plate for heating/DOP-modified PMP matrix and TiO_2_ NPs fillers in RC tape for cooling	multiple blade coating, esterification reactions	Cooling: Solar reflectance of > 90% and MIR absorptivity/emissivity of ~ 96%, average heating power of ~ 859.8 W m^−2^; Heating: Solar absorptivity of ~ 91%, average cooling power of ~ 126.0 W m^−2^	Intelligently auto-switched, zero-energy, scalable, and cost-effective	Weather resistance
Zhang et al. [72]	CNTs on the triacetate cellulose fibers	3D laser direct-write lithography printing	Modulate the IR radiation over 35%	Dyed, washable, and reversible gating effect	Costly fabrication

**Table 6:** Advantages and limitations of commonly used clothing fibers

Type	Fiber	Advantages	Limitations
Natural fiber	Cotton	Soft, breathable, warm, and moisture-absorbent	Easy to wrinkle (poor elasticity and stiffness), deform and shrink
	Linen	Cool and non-sticky feel when sweating; Natural luster, good color fastness, UV protection, and anti-mildew	Rough to feel, easy to wrinkle and shrink, and poor wearab comfort compared with cotton
	Silk	Soft and smooth, good luster and moisture absorption, and skin nourishing	Poor color fastness, bacterial and insect resistance. Easy to perish (rot) and generate static electricity
	Wool	Good elasticity, warmth, and moisture absorption, and antistatic property	Poor shape retention, bacterial and insect resistance. Easy to pill, shrink, and turn yellow
Synthetic fiber	Polyester	High strength and wearability. Good mildew, bacterial, wrinkle, and light resistance	Poor moisture absorption and air permeability, easy to pill, difficult to dye
	Nylon (Polyamide)	Good resilience and durability, light weight. Good mildew, bacterial, and solar radiation resistance	Poor moisture absorption, easy to wrinkle, poor heat and light resistance
	Acrylic (Polyacrylonitrile)	Good elasticity, high strength, and high warmth	Poor moisture absorption and wearability. Poor heat and alkali resistance
	Polypropylene	Light weight, good warmth, moisture absorption, and elasticity	Poor light and high-temperature resistance, easy to age
	Spandex (Polyurethane)	High elasticity and good shape retention	Poor moisture absorption, easy to break
